# Abnormal Paraventricular Nucleus of Hypothalamus and Growth Retardation Associated with Loss of Nuclear Receptor Gene *COUP*-*TFII*

**DOI:** 10.1038/s41598-017-05682-6

**Published:** 2017-07-13

**Authors:** Su Feng, Can Xing, Tingyu Shen, Yunbo Qiao, Ran Wang, Jun Chen, Jiaoyang Liao, Zhuo Lu, Xiong Yang, Saber Mohamed Abd-Allah, Jinsong Li, Naihe Jing, Ke Tang

**Affiliations:** 1 Institute of Life Science, Nanchang University, Nanchang, Jiangxi 330031 China; 2 State Key Laboratory of Cell Biology, CAS Center for Excellence in Molecular Cell Science, Shanghai Institute of Biochemistry and Cell Biology, Chinese Academy of Sciences; University of Chinese Academy of Sciences, 320 Yue Yang Road, Shanghai, 200031 China; 3 School of Life Science and Technology, ShanghaiTech University, 100 Haike Road, Shanghai, 201210 China; 4 Theriogenology Department, Faculty of Veterinary Medicine, Beni-Suef University, Beni-Suef, 62511 Egypt; 5 Precise Genome Engineering Center, School of Life Sciences, Guangzhou University, Guangzhou, 510006 China

## Abstract

The paraventricular nucleus of hypothalamus plays important roles in the regulation of energy balance and fetal growth. However, the molecular mechanisms underlying its formation and function have not been clearly elucidated. Various mutations in the human *COUP*-*TFII* gene, which encodes a nuclear receptor, result in growth retardation, congenital diaphragmatic hernia and congenital heart defects. Here, we show that *COUP*-*TFII* gene is expressed in the developing hypothalamus in mouse. The ventral forebrain-specific *RXCre*/+; *COUP*-*TFII*^*F*/*F*^ mutant mice display growth retardation. The development of the paraventricular nucleus of hypothalamus is compromised in the *COUP*-*TFII* mutant mainly because of increased apoptosis and mis-migration of the Brn2^+^ neurons. Moreover, hypoplastic anterior pituitary with blood cell clusters and shrunken posterior pituitary lacking AVP/OT neuron innervations are observed in the mutant, indicating the failure of formation of the hypothalamic-pituitary axis. Mechanistic studies show that the expression of *Bdnf* and *Nrp1* genes is reduced in the mutant embryo, and that *Bdnf* is a direct downstream target of the COUP-TFII protein. Thus, our findings provide a novel functional validation that *COUP*-*TFII* gene promotes the expression of *Bdnf* and *Nrp1* genes to ensure the appropriate morphogenesis of the hypothalamic-pituitary axis, especially the paraventricular nucleus of hypothalamus, and to prevent growth retardation.

## Introduction

The central nervous system, especially the hypothalamus, plays pivotal roles in the regulation of energy balance^1–3^. Several types of neurons in various murine hypothalamic nuclei have been identified to regulate food intake and energy expenditure, including AgRP and POMC neurons in the arcuate nucleus of hypothalamus^4–6^, oxytocin (OT) and melanocortin-4 receptor neurons in the paraventricular nucleus of hypothalamus (PVH)^7–10^, and Orexin and MCH neurons in the lateral hypothalamic area (LHA)^11–13^. Nevertheless, so far how these hypothalamic nuclei and molecularly defined neurons are generated remains largely unknown.

The PVH nucleus, which is located dorsally on either side of the third ventricle, participates in not only the regulation of energy balance but also the formation of the hypothalamic-pituitary (HP) axis^1,14^. There are two main groups of secretory neurons in the PVH nucleus: magnocellular neurons synthesizing the peptide hormones arginine vasopressin (AVP) or OT and parvocellular neurons secreting corticotropin-releasing hormone (CRH) or thyrotropin-releasing hormone (TRH)^15,16^. The magnocellular neurons in the PVH nucleus and the supraoptic nucleus (SON) project to the posterior pituitary, where AVP and OT are released. Several genes including *Brn2*, *Sim1*, *Otp* and *Arnt2* play important roles in the development of the PVH nucleus and posterior pituitary^17–24^. Growth retardation is observed in *Brn2*^−/−^, *Otp*^−/−^ and *Arnt2*^−/−^ mouse mainly because of the failure of formation of the HP axis^17,18,23,24^. Nonetheless, the molecular mechanism responsible for the appropriate formation of the HP axis, especially regarding the PVH nucleus, has not been fully understood.

Various rare deletions in chromosome 15q26 have been identified in patients with pre- and postnatal growth retardation, congenital heart defects (CHD), congenital diaphragmatic hernia (CDH), and high mortality^25–31^. *Chicken ovalbumin upstream promoter*-*transcription factor II* (*COUP*-*TFII*) gene (also known as *Arp*-*1*, *Nr2f2*, according to the Nuclear Receptors Nomenclature Committee 1999), mapped to 15q26.2, belongs to the steroid nuclear receptor superfamily^32^. Various copy number variants (CNVs) of the *COUP*-*TFII* gene have been identified in patients with growth restriction, CHD and CDH^25,27–30,33^. Especially, 11 out of 15 patients with *COUP*-*TFII* deletion have CDH^27,28,34^. One recent clinical study further demonstrated that several *de novo* single-nucleotide variants of *COUP*-*TFII* gene are responsible for the development of CHD^35^. *COUP*-*TFII* homozygous null mutant mouse is early embryonic lethal because of the failure of angiogenesis and heart development^36^. Conditional *COUP*-*TFII* homozygous mutant mouse with *Nkx3*.*2Cre* generates Bochdalek-type CDH^37^. The findings in mouse studies support the association of *COUP*-*TFII* with CHD and CDH. Consistent with the haploinsufficiency of *COUP*-*TFII* in patients, *COUP*-*TFII* gene heterozygous null mutant displays growth deficit and poor postnatal viability^36^. Nevertheless, so far how mutations of *COUP*-*TFII* gene cause growth failure remains unknown.

Here, we found that *COUP*-*TFII* is expressed in the developing hypothalamus of mouse. Ventral forebrain-specific *RXCre*/+; *COUP*-*TFII*^*F*/*F*^ homozygous mutant mice generate postnatal growth retardation and poor viability. Hypocellular PVH and SON nuclei are observed in the mutants, which may be caused by increased apoptosis and mis-migration of the Brn2^+^ neurons. In addition, hypoplastic anterior pituitary with blood cell clusters and shrunken posterior pituitary lacking AVP/OT neuron projection are observed in the mutant. Furthermore, the expression of *Bdnf*, *Nrp1* and *Avp* genes is reduced in the ventral forebrain of the mutant, and *Bdnf* is a direct downstream target of the COUP-TFII protein.

## Results

### *COUP*-*TFII* gene is expressed in the developing hypothalamus of mouse

**Figure 1:**
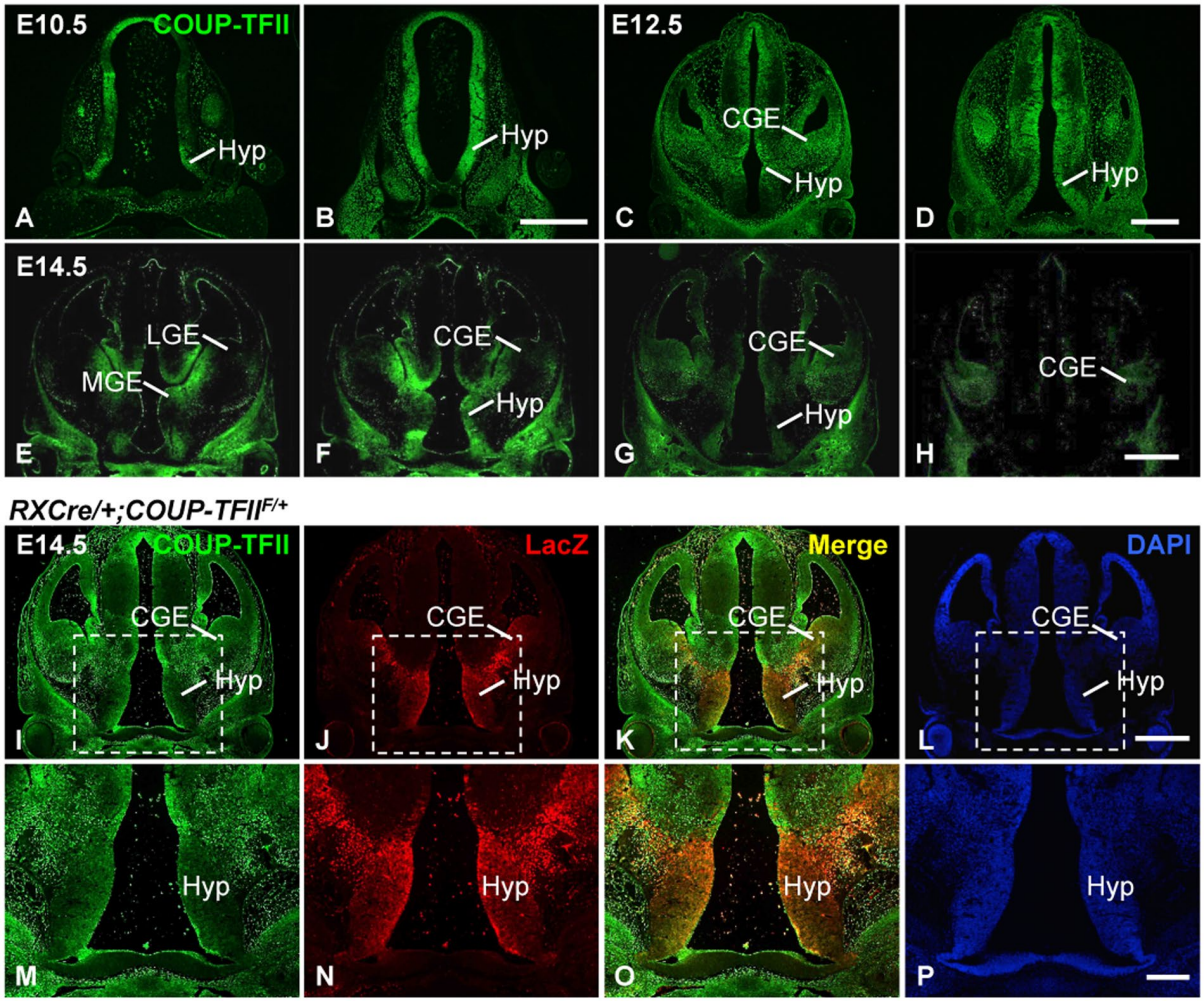
Expression of *COUP*-*TFII* in the developing hypothalamus and the RXCre recombinase activity in the hypothalamus. The expression of COUP-TFII protein is detected in neuronal progenitor cells at the ventricular zone of the hypothalamus at E10.5 (**A**,**B**), in the hypothalamic region at E12.5 (**C**,**D**) and E14.5 (**E**–**H**). (**I**–**P**) COUP-TFII (green) and LacZ (red) are colocalized at the hypothalamus of the *RXCre*/+; *COUP*-*TFII*^*F*/+^ heterozygous mutant at E14.5. (**M**–**P**) Inserts in (**I**–**L**). CGE, caudal ganglionic eminence; Hyp, Hypothalamus; LGE, lateral ganglionic eminence; MGE, medial ganglionic eminence. Two or three embryos were analyzed at each stage. Scale bars, (**A**–**D**,**E**–**H**,**I**–**L**) 500 μm; (**M**–**P**) 200 μm.

Since patients with various 15q26 deletions including the *COUP*-*TFII* gene display growth retardation and *COUP*-*TFII* gene heterozygous mutant mice generate developmental delay and poor postnatal viability^36^, we asked whether *COUP*-*TFII* in the hypothalamus contributes to the regulation of growth deficit. To answer this question, we first assessed the expression of *COUP*-*TFII* in mouse embryonic forebrain by immunofluorescence assays. At E10.5, the expression of COUP-TFII was detected in the ventricular zone of the hypothalamus, where reside neuronal progenitor cells (NPCs) (Fig. 1A,B). The expression of COUP-TFII was confined to the hypothalamic region at E12.5 (Fig. 1C,D) and E14.5 (Fig. 1F,G). Thus, COUP-TFII is expressed in the NPCs and early differentiating neurons of the mouse hypothalamus.

It has been shown that the activity of RXCre recombinase is detected in the mouse embryonic ventral forebrain including the eye and the hypothalamus^38^, and LacZ expression can be used as an indicator for the deletion of *COUP*-*TFII* gene^39^. We performed double immunofluorescence staining assays with antibodies against COUP-TFII and LacZ on coronal sections of a *RXCre*/+; *COUP*-*TFII*^*F*/+^ heterozygous mutant embryo at E14.5. COUP-TFII was expressed at the hypothalamus (Fig. 1I,M). The expression of LacZ was also detected at the hypothalamus and the caudal ganglionic eminence (Fig. 1J,N). Merged images revealed that the green COUP-TFII signals and the red LacZ signals were highly colocalized at the hypothalamus (Fig. 1K,O), suggesting that RXCre recombinase can efficiently excise the *COUP*-*TFII* gene in the embryonic hypothalamus.

### *RXCre*/+; *COUP*-*TFII*^*F*/*F*^ mutant mice display growth retardation and compromised PVH nucleus

**Figure 2:**
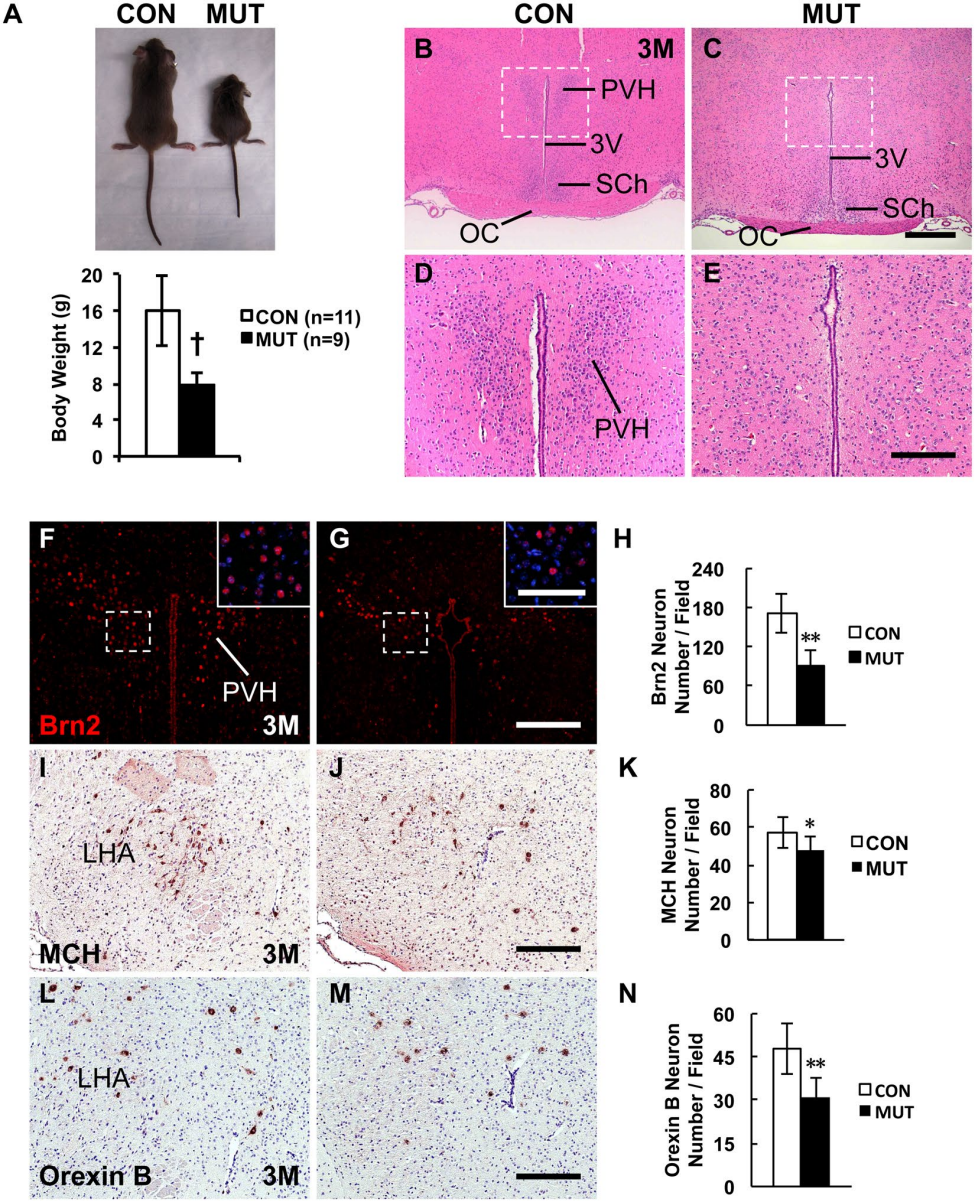
Adult *COUP*-*TFII* gene mutant mice with *RXCre* display growth retardation and compromised PVH nucleus. (**A**) Body weight of the *RXCre*/+; *COUP*-*TFII*^*F*/*F*^ male mutant mice (n = 6) is approximately half of that of the control (n = 8) at wean. The H&E staining data reveal that compared with the control (**B**,**D**), the PVH nucleus is barely detectable in the mutant at 3 M (**C**,**E**). (**D**,**E**) Inserts in (**B**,**C**). Compared with the control (**F**), there are very few Brn2^+^ PVH neurons in the mutant at 3 M (**G** and insert), and the reduction is significant (**H** and insert). Compared with the control (**I**), there are fewer MCH neurons in the mutant LHA region at 3 M (**J**), and the reduction is significant (**K**). Compared with the control (**L**), there are fewer Orexin B neurons in the mutant LHA region at 3 M (**M**), and the reduction is significant (**N**). 3 V, third ventricle; LHA, lateral hypothalamic area; OC, optic chiasm; PVH, paraventricular nucleus of hypothalamus; SCh, suprachiasmatic nucleus. The quantitative data in (**H**,**K** and **N**) were generated from the analysis of three pairs of control and mutant mice. The data indicate the mean ± SD. Student’s t-test, *P < 0.05; **P < 0.01; ^†^P < 0.001. Scale bars, (**B**,**C**) 500 μm; (**D**,**E**,**F**,**G**) 200 μm; inserts of (**F**,**G**) 100 μm; (**I**,**J**,**L**,**M**) 100 μm.

*RXCre* mouse was used to generate the *RXCre*/+; *COUP*-*TFII*^*F*/*F*^ conditional homozygous mutant mouse, referred to as *COUP*-*TFII* mutant or mutant hereafter. At birth, all the pups in the same litter were similar. Nevertheless, the mutant pups were smaller than their control littermates at postnatal day 3 (P3) and day 4 (P4) (data not shown). Some mutant mice did not survive between P18 and P26, and some survived to adulthood. No obvious differences were observed among *COUP*-*TFII*^*F*/+^, *COUP*-*TFII*^*F*/*F*^ and *RXCre*/+; *COUP*-*TFII*^*F*/+^ mice; therefore, they were used as the control in the study. The body weight of the mutant mice was only approximately half that of the control mice at wean (Fig. 2A). Clearly, *RXCre*/+; *COUP*-*TFII*^*F*/*F*^ homozygous mutant mice displayed growth retardation.

To investigate the cause of growth restriction, we performed H&E staining on coronal sections from the control and the mutant mouse at 3 weeks (3 W) and 3 months (3 M) after birth. The same phenotypes were observed at both stages, and images generated from mice at 3 M were shown. Compared with the control (Fig. 2B,D), the PVH nucleus was barely observed in the mutant (Fig. 2C,E). *Brn2* gene is specifically expressed in the PVH neurons^17,18^. Immunofluorescence staining showed that compared with the control (Fig. 2F and insert), there were much fewer Brn2^+^ PVH neurons in the mutant (Fig. 2G and insert). Quantitative analysis from three pairs of mice showed that the reduction of Brn2^+^ PVH neurons in the mutant was significant (Fig. 2H). Overall, therefore, the development of the PVH nucleus is morphologically and molecularly compromised in the *COUP*-*TFII* mutant mouse.

MCH neurons and Orexin B neurons in the LHA promote food intake, and *MCH* mutant or Orexin B neuron-ablating mouse displays hypophagia^11–13^. Compared with the control (Fig. 2I), the number of MCH neurons was significantly reduced in the mutant LHA (Fig. 2J,K), as was the number of Orexin B neurons in the mutant (Fig. 2L–N). POMC and NPY neurons in the arcuate nucleus also participate in the regulation of energy balance^4–6^. The expression of POMC and NPY in the arcuate nucleus was comparable between the control and the mutant mouse (data not shown). GHRH neurons in the arcuate nucleus control body growth through regulating growth hormone pathway^40–42^. Compared with the control (Fig. S1A,C), there were much few GHRH neurons in the arcuate nucleus in the mutant at 1 M (Fig. S1B,D), and the reduction was significant (Fig. S1E). Similar as the *RXCre*/+; *COUP*-*TFII*^*F*/+^ control mouse (Fig. S1F,H), the expression of LacZ was barely detected in the arcuate nucleus in the *RXCre*/+; *COUP*-*TFII*^*F*/*F*^ mutant mouse (Fig. S1G,I), indicating *COUP*-*TFII* gene is not deleted in the mutant arcuate nucleus. The data above suggest that the reduced MCH, Orexin B and GHRH neurons in the hypothalamic regions may contribute to growth defect in the *COUP*-*TFII* mutant.

**Figure 3:**
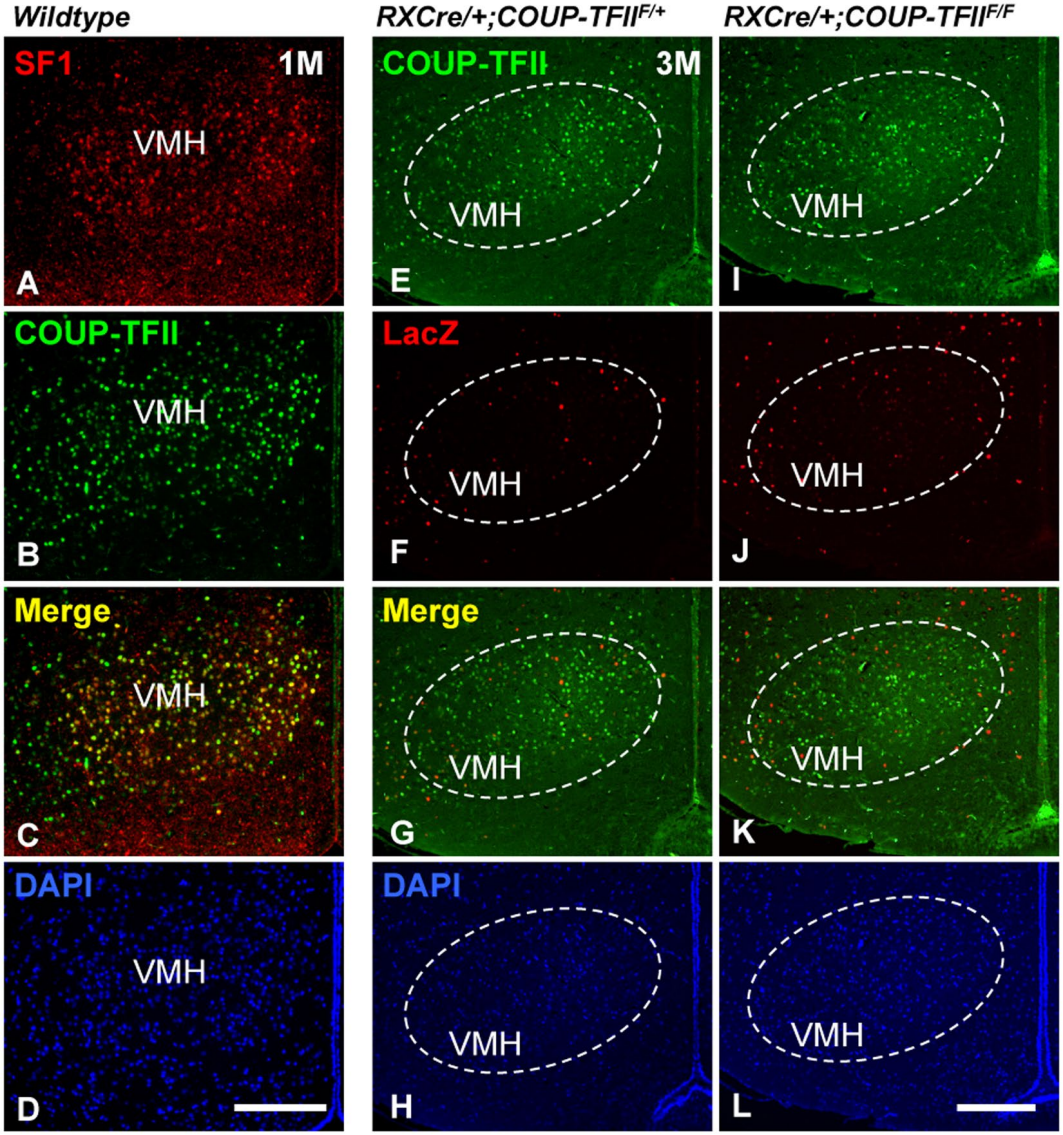
Development of the VMH nucleus is normal in the adult *COUP*-*TFII* mutant. (**A**–**D**) The expression of COUP-TFII (green) is readily detected in the majority of SF1^+^ VMH neurons (red) at 1 M. (**E**–**L**) The expression of COUP-TFII (green) in the VMH nucleus was not altered between the control and the mutant at 3 M; additionally, there were few LacZ signals (red) in the VMH nucleus of the control and the mutant. VMH, ventromedial nucleus of hypothalamus. Scale bars, (**A**–**D**,**E**–**L**) 200 μm.

The expression of *COUP*-*TFII* in the ventromedial nucleus of hypothalamus (VMH) of mouse is related to hypoglycemia-associated autonomic failure^43^. Consistent with a previous report^43^, the expression of COUP-TFII was readily detected in the majority of SF1^+^ VMH neurons at 1 M (Fig. 3A–D). Double immunostaining with antibodies against COUP-TFII and LacZ was conducted in the *RXCre*/+; *COUP*-*TFII*^*F*/+^ heterozygous and *RXCre*/+; *COUP*-*TFII*^*F*/*F*^ homozygous mice at 3 M. The expression of both COUP-TFII and LacZ in the VMH nucleus was similar between the control and the mutant mouse (Fig. 3E–L). It seems that RXCre recombinase does not target the VMH neurons, and the development of the VMH nucleus is normal in the *COUP*-*TFII* mutant mouse. Thus, in the mutant hypothalamus, the differentiation of Brn2^+^ neurons in the PVH nucleus, MCH and Orexin B neurons in the LHA was affected, but not POMC and NPY neurons in the arcuate nucleus and COUP-TFII^+^ neurons in the VMH nucleus.

### COUP-TFII is expressed in Brn**2**^+^ early differentiating PVH neurons

**Figure 4:**
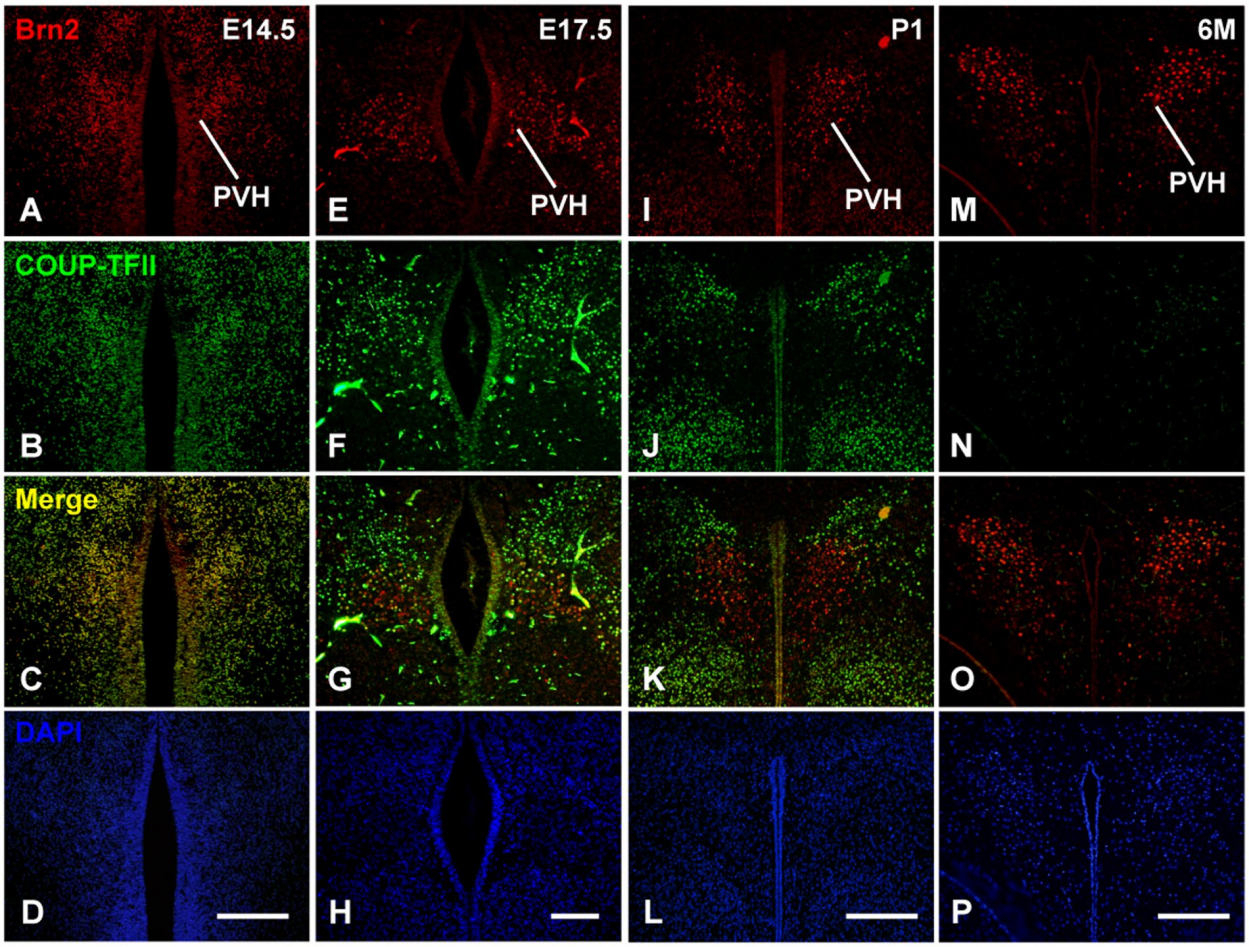
COUP-TFII is co-expressed with Brn2 in the early differentiating PVH neurons, but not in the late differentiating PVH neurons and the mature PVH neurons. (**A**–**D**) Colocalization of Brn2 (red) and COUP-TFII (green) in the early differentiating PVH neurons at E14.5. (**E**–**H**) Expression of Brn2 and COUP-TFII in the differentiating PVH neurons at E17.5. (**I**–**L**) Expression of Brn2 and COUP-TFII in the late differentiating PVH neurons at P1. (**M**–**P**) Expression of Brn2 and COUP-TFII in the mature PVH neurons at 6 M. PVH, paraventricular nucleus of hypothalamus. Scale bars, (**A**–**D**,**I**–**L**,**M**–**P**) 200 μm; (**E**–**H**) 100 μm.

Hypocellular PVH nucleus is the most obvious defect observed in the *COUP*-*TFII* mutant (Fig. 2B–H). It has been reported that *Brn2* gene plays a pivotal role in the differentiation of the PVH neuron^17,18^. We performed double immunofluorescence staining with antibodies against COUP-TFII and Brn2 on coronal sections containing the PVH nucleus at E14.5, E17.5, P1 and 6 M. The expression of Brn2 was detected at the prospective PVH nucleus at E14.5 (Fig. 4A), in the late differentiating PVH neuron at E17.5 (Fig. 4E) and at P1 (Fig. 4I), and in the mature PVH neurons at 6 M (Fig. 4M). The expression of COUP-TFII was broadly detected in the hypothalamic regions, and was colocalized with Brn2 in the early differentiating PVH neurons at E14.5 (Fig. 4B–D). At E17.5, the expression of COUP-TFII was sharply decreased in the Brn2^+^ differentiating PVH neurons, but remained high in the neurons localized dorsally to the PVH nucleus (Fig. 4F–H). The expression of COUP-TFII was barely detectable in the Brn2^+^ late differentiating PVH neurons at P1 (Fig. 4J–L) and the Brn2^+^ mature PVH neurons at 6 M (Fig. 4N–P). These data show that *COUP*-*TFII* gene is expressed in the early differentiating PVH neurons, but not in the late differentiating and the mature PVH neurons.

### Reduced Brn**2**^+^ early differentiating PVH neurons, hypocellular SON nucleus, and increased apoptosis in the *COUP*-*TFII* mutant embryo

**Figure 5:**
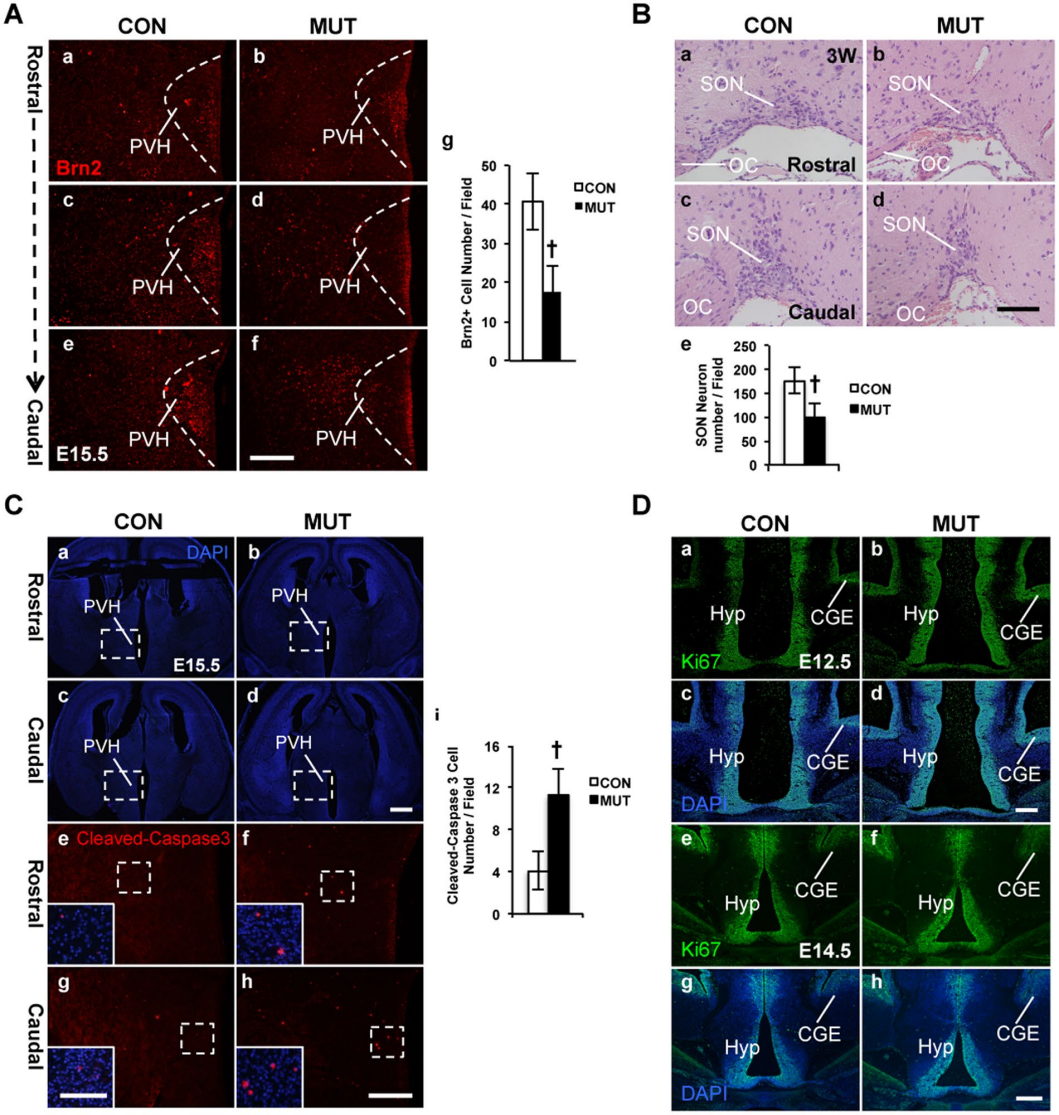
Reduction of the Brn2^+^ early differentiating PVH neurons, hypocellular SON nucleus, and increased apoptosis in the *COUP*-*TFII* mutant. (**A**) Brn2^+^ early differentiating PVH neurons are reduced in the mutant embryo. Compared with the control (**A**a–c), there are much fewer Brn2^+^ early differentiating PVH neurons along the rostro-caudal axis in the mutant at E15.5 (**A**d–f), and the reduction is significant (**A**g). The data are collected from three pairs of control and mutant embryos. (**B**) Development of the SON nucleus is abnormal in the adult mutant mouse. The H&E staining data show that compared with the control (**B**a,b), there are much fewer SON neurons in the *COUP*-*TFII* mutant (**B**c,d). The reduction of magnocellular SON neurons is significant in the mutant (**B**e). (**C**a–h) Increased apoptosis is detected in the *COUP*-*TFII* mutant embryo. DAPI staining images of coronal sections with PVH region in the control (**C**a,c) and the mutant (**C**b,d) at E15.5. Compared with the control (**C**e,g and inserts), there are more cleaved-Caspase-3 signals in the mutant PVH region at E15.5 (**C**f,h and inserts), and the increase of the cleaved-Caspase-3 signals is significant (**C**i). (**D**a–h) Proliferation is not affected in the *COUP*-*TFII* mutant. The expression of Ki67 is comparable between the control (**D**a,c) and mutant (**D**b,d) at E12.5. Expression of Ki67 is comparable between the control (**D**e,g) and mutant (**D**f,h) at E14.5. CGE, caudal ganglionic eminence; OC, optic chiasm; PVH, paraventricular nucleus of hypothalamus; SON, supraoptic nucleus. At least three pairs of the control and the mutant mice were used in each study. The data indicate the mean ± SD. Student’s t-test, ^†^P < 0.001. Scale bars, (**A**a–f) 200 μm; (**B**a–d) 100 μm; (**C**a–d) 500 μm; (**C**e–h,**D**a–h) 100 μm; inserts of (**C**e–h) 50 μm.

Next, we asked whether the development of the PVH nucleus is normal at embryonic stages. As shown in Fig. 5Aa,c,e, there were many Brn2^+^ neurons at the PVH region along the rostro-caudal axis in the control at E15.5. In contrast, there were very few Brn2^+^ neurons at the prospected mutant PVH nucleus (Fig. 5Ab,d,f). Quantitative data from three pairs of animals at the same stage revealed that compared with the control, the reduction of the Brn2^+^ early differentiating PVH neurons was significant in the mutant (Fig. 5Ag).

Interestingly, many Brn2^+^ neurons were localized laterally to the prospected caudal PVH nucleus in the mutant (Fig. 5Ad,f). Calbindin is another marker of the SON and PVH neurons^17,44^. Similar to the Brn2^+^ neurons, compared with the control (Fig. S2A,C,E,G), there were more mis-migrating Calbindin^+^ neurons beside the caudal PVH nucleus in the mutant (Fig. S2B,D,F,H). The SON nucleus lies lateral to optic tracts and dorsal to pial surface of the brain. Neurons in the PVH nucleus and the SON nucleus originate from the same group of NPCs at E10.5^45^. During development, the PVH neurons are differentiated locally, while the SON neurons migrate ventro-laterally to their final destination^45^. Probably, those mis-located Brn2^+^ or Calbindin^+^ neurons are related to the SON neurons. As expected, H&E staining results revealed that compared with the control (Fig. 5Ba,c), the number of magnocellular SON neurons in the mutant was reduced (Fig. 5Bb,d), and the reduction was significant (Fig. 5Be). Thus, the formation of the SON nucleus is also affected in the *COUP*-*TFII* mutant.

There are several possibilities for the loss of the Brn2^+^ PVH neurons in the *COUP*-*TFII* mutant embryos, such as apoptosis and proliferation defect. To investigate the mechanism for the reduction of the Brn2^+^ PVH neurons, we performed immunostaining assay to examine the expression of cleaved-Caspase-3, an apoptotic marker. Compared with the control at E15.5 (Fig. 5Ca,c,e,g, and inserts), there were more cleaved-Caspase-3 signals in the mutant PVH region (Fig. 5Cb,d,f,h, and inserts). Quantitative assays with samples from 3 pairs of animals at E15.5 confirmed that there were significantly more apoptotic cells in the mutant PVH than in the control (Fig. 5Ci). In addition, there were also more cleaved-Caspase-3 signals in the hypothalamus of the mutant than the control at E13.5 (Fig. S3A–F), indicating that abnormal apoptosis occurs at earlier embryonic stages. Next, we assessed the expression of Ki67, a proliferation marker, at the PVH region at E12.5 and at E14.5. The expression of Ki67 was comparable between the control (Fig. 5Da,c,e,g) and the mutant (Fig. 5Db,d,f,h) at both stages, suggesting that proliferation was not altered in the mutant. Thus, our data show that increased apoptosis is one possible mechanism that leads to the reduction of the Brn2^+^ PVH neurons in the *COUP*-*TFII* mutant.

### *Bdnf* gene is a direct downstream target of COUP-TFII

**Figure 6:**
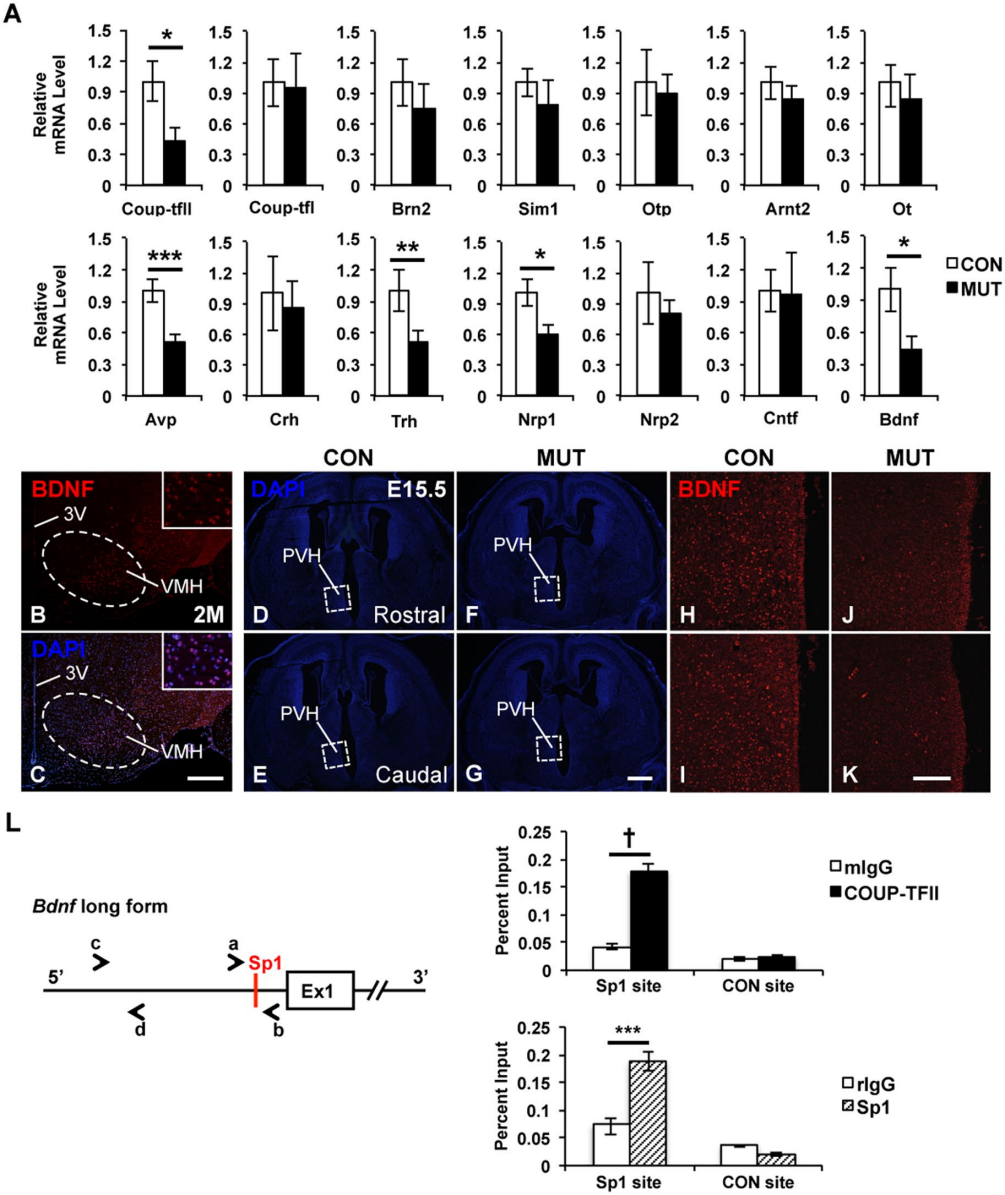
Expression of *Bdnf* and *Nrp1* is reduced in the *COUP*-*TFII* mutant embryo, and *Bdnf* is a downstream target of COUP-TFII. (**A**) Real-time quantitative PCR data with samples from the ventral forebrain of the control (n = 4) and mutant (n = 3) embryos at E14.5. The expression of *COUP*-*TFII*, *Avp*, *Bdnf*, *Nrp1* and *Trh* transcripts is significantly reduced in the mutant. A BDNF-specific antibody detects the expression of BDNF in the cytoplasm of neurons at the ventro-lateral region of the mouse VMH nucleus at 2 M (**B**,**C**). DAPI staining images of coronal sections with the PVH region in the control (**D**,**E**) and the mutant (**F**,**G**) at E15.5. Compared with the control (**H**,**I**), the expression of BDNF protein is noticeably reduced in the prospected mutant hypothalamus at E15.5 (**J**–**K**). L, An evolutionarily conserved Sp1 binding site is identified at the promoter region of the long form of the *Bdnf* gene. Primers a/b and c/d were used to amplify the Sp1 locus and a 2 kb up-stream non-Sp1 control locus, respectively. In chromatin immunoprecipitation assays, the binding of both COUP-TFII and Sp1 protein is enriched at the conserved Sp1 site but not at the negative control site. 3 V, third ventricle; PVH, paraventricular nucleus of hypothalamus; VMH, ventromedial nucleus of hypothalamus. Three independent assays were performed in each real-time quantitative PCR experiment. The data indicate the mean ± SD. Student’s t-test, *P < 0.05; **P < 0.01; ***P < 0.005; ^†^P < 0.001. Scale bar, (**B**,**C**) 300 μm; (**D**–**G**) 500 μm; (**H**–**K**) 100 μm.

To investigate molecular mechanism responsible for the defects in the *COUP*-*TFII* mutant, real-time quantitative PCR (*qPCR*) assays were performed with total RNAs prepared from ventral forebrains of the control and the mutant at E14.5. As shown in Fig. 6A, the expression of *COUP*-*TFII* transcripts was reduced by approximately 50% in the mutant compared with the control. The expression of the *COUP*-*TFI* gene was not altered. *Brn2*, *Sim1*, *Otp* and *Arnt2* genes are essential for the development of the PVH nucleus^17–23^. Compared with the control, the expression of these genes was slightly reduced in the mutant mouse at E14.5, but the difference was not significant (Fig. 6A), indicating that the function of *COUP*-*TFII* in the development of the PVH nucleus may be independent of these known regulatory genes.

There are several major secretory neurons in the PVH nucleus including the AVP, OT, CRH and TRH neurons^15,16^. Interestingly, the expression of *Avp* and *Trh* genes but not *Ot* and *Crh* genes was significantly reduced in the mutant at E14.5 (Fig. 6A), suggesting that *COUP*-*TFII* gene may be specifically required for the early differentiation of the AVP and TRH neurons. *Nrp1* and *Nrp2* genes, which are involved in cell migration and axon guidance, are regulated directly by COUP-TFII protein^39^. The expression of both *Nrp1* and *Nrp2* genes is reduced in the ventral forebrain of *RXCre*/+; *COUP*-*TFII*^*F*/*F*^ mutant mouse at E12.5^39^. The expression of *Nrp1* but not *Nrp2* was decreased significantly in the mutant at E14.5 (Fig. 6A), suggesting that *Nrp1* might specifically mediate the migration of the Brn2^+^ neurons in the hypothalamus.

Neurotrophin factors, such as NGF, BDNF, NT3 and CNTF, are essential for the survival and differentiation of neurons^46–48^. Since the increased apoptosis was detected in the mutant hypothalamus (Figs 5C and S3), the expression of neurotrophin factors was assessed by *qPCR* assays. The expression of the *Ngf* and *Nt3* genes was undetectable in both the control and the mutant (data not shown). The expression of *Cntf* transcripts was not altered in the mutant, whereas the expression of *Bdnf* was significant lower in the mutant than the control (Fig. 6A). *Bdnf* transcripts are preferentially expressed at the ventro-lateral region of the VMH nucleus^49^. A specific BDNF antibody could detect the expression of BDNF protein in the cytoplasm of neurons at the ventro-lateral VMH nucleus in the adult mouse at 2 M (Fig. 6B,C). The expression of BDNF was readily detected in the rostral and the caudal part of the control hypothalamic areas including the PVH nucleus at E15.5 (Fig. 6D,E,H,I); in contrast, its expression was barely detectable in the mutant (Fig. 6F,G,J,K). Thus, the expression of *Bdnf* was reduced in the *COUP*-*TFII* mutant at both transcriptional and translational levels.

To determine the direct downstream targets of COUP-TFII among the *Avp*, *Bdnf*, *Nrp1* and *Trh* genes, chromatin immuoprecipitation (*ChIP*) assay was performed with chromatin prepared from the hypothalamus of mouse embryos at E14.5. COUP-TFII positively regulates the expression of target genes through the Sp1 site by tethering to Sp1 protein^32,50,51^. COUP-TFII may promote the expression of *Nrp1* gene in the ventral forebrain through a conserved Sp1 site in intron 12^39^. Our *ChIP*-*qPCR* assays showed that both COUP-TFII and Sp1 were recruited at the conserved Sp1 site of *Nrp1* gene, but not at the control non-Sp1 site at 3′ UTR (Fig. S4). Next, an Sp1 site, which is evolutionarily conserved among human, chimpanzee, mouse, cow and opossum, was identified at the promoter region of the long form of the *Bdnf* gene (Fig. 6L). The binding of both COUP-TFII and Sp1 was enriched at the conserved Sp1 site of *Bdnf* gene, but not at a negative non-Sp1 control site, which was located 2 kb upstream (Fig. 6L). Unfortunately, no such evolutionarily conserved Sp1 site was identified in the genomic locus of either *Avp* or *Trh*. The findings above suggest that the *Bdnf* gene is a direct downstream target of COUP-TFII, and the reduced expression of *Bdnf* is a possible cause for the increased apoptosis in the *COUP*-*TFII* mutant.

### The development of pituitary is compromised in the *COUP*-*TFII* mutant

**Figure 7:**
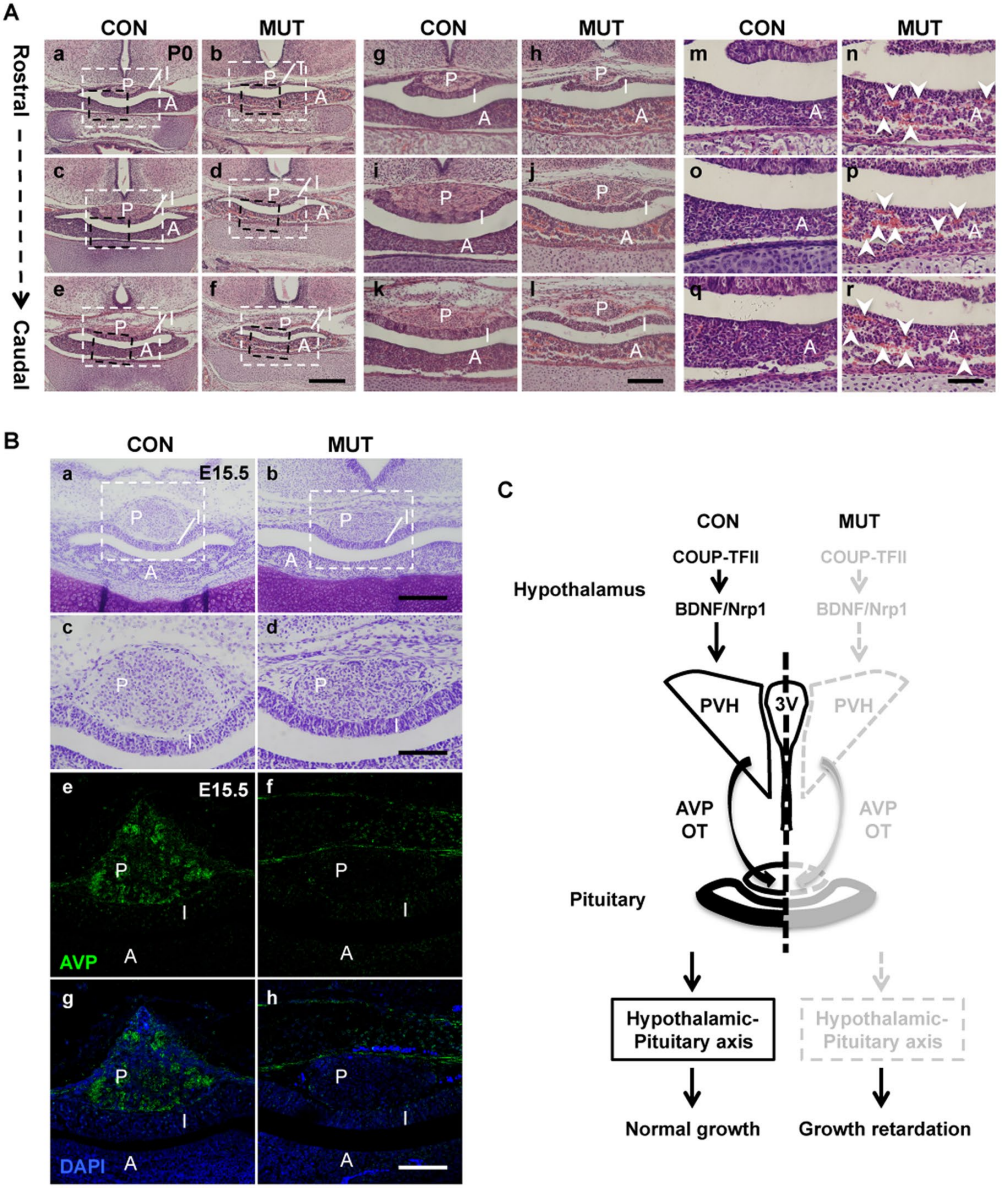
*COUP*-*TFII* ensures the appropriate morphogenesis and function of the hypothalamic-pituitary axis, especially the PVH nucleus, to prevent growth retardation. (**A**), The development of both posterior and anterior pituitary is abnormal in the newborn mutant. (**A**g–i) White inserts in (**A**a–f). (**A**m–r) Black inserts in (**A**a–f). H&E staining showed that compared with the control (**A**a,c,e,g,i,k), the posterior pituitary is shrunken along the rostro-caudal axis in the mutant at P0 (**A**b,d,f,h,j,l). There are a few isolated blood cells in the anterior pituitary of the control (**A**m,o,q); however, many blood cell clusters, indicated by white arrow-heads, are observed in the mutant anterior pituitary (**A**n,p,r). (**B**) The projection of AVP neuron to the posterior pituitary is abnormal in the mutant embryo at E15.5. Nissl staining images of the pituitary in the control (**B**a,c) and the mutant at E15.5 (**B**b,d). (**B**c,d) Inserts in (**B**a,b). The expression of AVP is readily detected in the control posterior pituitary at E15.5 (**B**e,g), but not in the mutant posterior pituitary (**B**f,h). (**C**) A working model: during early development, *COUP*-*TFII* may activate or maintain the expression of *Bdnf* and *Nrp1* genes to ensure the appropriate morphogenesis and function of the hypothalamic-pituitary axis, especially the PVH nucleus, and to prevent the growth failure. 3 V, third ventricle; A, anterior pituitary lobe; I, intermediate pituitary lobe; P, posterior pituitary lobe; PVH, paraventricular nucleus of hypothalamus. Scale bar, (**A**a–f) 400 μm; (**A**g–l) 200 μm; (**A**m–r) 100 μm; (**B**a,b) 200 μm; (**B**c–h) 100 μm.

In *Brn2*^−/−^ null mutant mouse, the absence of the PVH and SON nuclei leads to the loss of the posterior pituitary, failure of formation of the HP axis, and growth retardation^17,18^. The pituitary gland consists of anterior (A), intermediate (I) and posterior (P) pituitary lobes (Fig. 7A). H&E staining results revealed that compared with the control (Fig. 7Aa,c,e,g,i,k), the posterior pituitary was noticeably smaller in the *COUP*-*TFII* mutant at P0 (Fig. 7Ab,d,f,h,j,l). Moreover, images at higher magnification revealed that there were a few isolated blood cells in the anterior pituitary of the control (Fig. 7Am,o,q); nevertheless, many blood cell clusters, indicated by white arrowheads, were observed in the anterior pituitary of the mutant (Fig. 7n,p,r), indicating a hypoplastic anterior pituitary. Most likely, the defective anterior and posterior pituitary is a cause of growth restriction of the *COUP*-*TFII* mutant.

Axon projections of AVP or OT magnocellular neurons target the posterior pituitary between E15.5 and E16.5^52^. The expression of AVP was readily detected in the control at E15.5 (Fig. 7Be,g); however, its expression was absent from the posterior pituitary of the mutant (Fig. 7Bf,h). In addition, compared with the control (Fig. S5A,C,E,G), the expression of both AVP and OT was barely detected in the mutant posterior pituitary at P0 (Fig. S5B,D,F,H). In the *COUP*-*TFII* mutant mouse, hypocellular PVH/SON nuclei, hypoplastic anterior pituitary, and shrunken posterior pituitary lead to the failure of formation of the HP axis, which could be a main reason for growth retardation of these mice.

## Discussion

In the present study, we have observed that *COUP*-*TFII* is expressed in the developing embryonic hypothalamus. Similar to the phenotype of hemizygous deletion of *COUP*-*TFII* in both human and mouse, *RXCre*/+; *COUP*-*TFII*^*F*/*F*^ mutant mice display noticeable growth restriction and poor postnatal viability. The development of the PVH and SON nuclei is affected in the *COUP*-*TFII* mutant mouse, with defects in both the anterior and the posterior pituitary. Moreover, increased apoptosis and mis-migration of the Brn2^+^ neurons are observed in the mutant. Mechanistic studies reveal that *COUP*-*TFII* may maintain or activate the expression of *Bdnf* and *Nrp1* genes to ensure the appropriate morphogenesis and function of the HP axis, especially the PVH nucleus.

All patients with various 15q26 deletions generate growth retardation, associated with CDH and/or CHD^25–31^. The evidence from both clinical and mouse studies suggests that among the genes at the 15q26 locus, the loss of *IGF1R* at 15q26.3 is responsible for pre- and postnatal growth restriction^31,53–55^. Interestingly, various CNVs of the *COUP*-*TFII* gene were also identified in patients with different 15q26 deletions^25,27–30,33^. Moreover, the loss of an allele of the *COUP*-*TFII* gene leads to growth retardation and poor postnatal viability in pure-bred 129 Sv, C57BL/6 or ICR mouse and in 129 Sv/C57BL/6 mixture mouse^36^, indicating complete penetrance of the defect. Here, we find that the *RXCre*/+; *COUP*-*TFII*^*F*/*F*^ mutant mice are half the size of the control mice at wean (Fig. 2), suggesting that the *RXCre*/+; *COUP*-*TFII*^*F*/*F*^ mutant mouse phenocopies the *COUP*-*TFII* heterozygous null mouse. Therefore, other than *IGF1R*, haploinsufficiency of *COUP*-*TFII* is a possible cause for growth deficit in patients with 15q26 deletions.

The HP axis is an important neuroendocrine system coordinating the periphery and brain signals required for physiologic homeostasis and survival^16,56^. However, the detailed molecular and cellular mechanism responsible for the formation of the HP axis has not been fully clarified. Because the *COUP*-*TFII* gene is not expressed in the pituitary gland^57^, we mainly focused on its function in the hypothalamus. *COUP*-*TFII* is expressed in the hypothalamic NPCs and early differentiating PVH neurons, but not in the late differentiating and the mature PVH neurons (Figs 1 and 4), suggesting that *COUP*-*TFII* may play crucial roles in the early development of the hypothalamus. Indeed, the morphogenesis of the PVH nucleus is compromised in the *COUP*-*TFII* mutant with hypocellular SON nucleus, hypoplastic anterior pituitary with blood cell clusters, and shrunken posterior pituitary lacking AVP/OT projections (Figs 2, 5 and 7 and S5). The *COUP*-*TFII* mutant mouse phenocopies *Brn2*^−/−^, *Sim1*^−/−^ and *Arnt2*^−/−^ mouse in terms of growth restriction and compromised posterior pituitary^17–23^. However, the development of the anterior pituitary is not affected in the *Brn2*^−/−^, *Sim1*^−/−^ and *Arnt2*^−/−^ mouse^17–23^. In contrast, hypoplastic anterior pituitary is detected in the *COUP*-*TFII* mutant (Fig. 7), suggesting that the failure of formation of the HP axis, especially a defective anterior pituitary, is a possible cause for growth failure of the *COUP*-*TFII* mutant. The hypoplastic anterior pituitary could be caused by the reduction of GHRH neurons in the arcuate nucleus in the mutant (Fig. S1). The expression of *Brn2*, *Sim1*, *Otp* and *Arnt2* transcripts is comparable between the control and the mutant (Fig. 6). The development of PVH NPCs remains normal in either *Brn2*^−/−^ or *Sim1*^−/−^ null mutant mouse at E15.5^17–19^. Nonetheless, the Brn2^+^ early differentiating PVH neurons are noticeably reduced in the *COUP*-*TFII* mutant at E15.5 (Fig. 5). Therefore, distinct from *Brn2* and *Sim1*, which participate in the terminal differentiation of PVH neurons, *COUP*-*TFII* is a novel key regulatory gene mediating the early morphogenesis of the HP axis, especially the PVH nucleus.

A hypocellular PVH nucleus is one of the most significant phenotypes in the *COUP*-*TFII* mutant (Fig. 2), which may be caused by several possibilities including abnormal apoptosis, lower proliferation, mis-migration or inappropriate differentiation. The Ki67 staining data show that proliferation is not altered in the mutant (Fig. 6). Both the PVH and SON neurons originate from the same population of NPCs at E10.5^45^. A few Brn2^+^ or Calbindin^+^ neurons are mis-located laterally to the prospective mutant PVH nucleus (Figs 5 and S2), indicating that mis-migration may contribute to the hypocelluar PVH and SON nuclei in the mutant. As the direct targets of COUP-TFII protein, the *Nrp1* and *Nrp2* genes mediate the migration of Pax6^+^ neurons from the caudal ganglionic eminence to the basal medial amygdala nucleus^39^. Consistently, the expression of *Nrp1* transcripts is reduced in the mutant at E14.5 (Fig. 6), suggesting that *Nrp1* may specifically participate in the regulation of migration of the Brn2^+^ neuron in the hypothalamus. Furthermore, the number of cleaved-Caspase-3^+^ apoptotic cells is significantly increased in the mutant (Fig. 5). Neurotrophins, such as NGF and BDNF, are essential for the survival and growth of neurons during development^46,47,58^. Intriguingly, *Bdnf*^−/−^ mouse displays postnatal growth retardation^59^; however, the cause of growth restriction in *Bdnf*^−/−^ mouse is not clear. In the *COUP*-*TFII* mutant embryo, the expression of *Bdnf* is reduced at both the transcriptional and translational levels (Fig. 6). *ChIP* assay *in vivo* data demonstrate further that the *Bdnf* gene is a direct downstream target of COUP-TFII (Fig. 6). RXCre recombinase deletes the *COUP*-*TFII* gene in the ventral forebrain including the hypothalamus and caudal ganglionic eminence (Fig. 1)^39^. The reduced expression of *Bdnf* protein is also observed in the ventral forebrain regions of the mutant (Fig. 6), which may explain the reduction of the Brn2^+^ PVH neurons, as well as MCH neurons and Orexin B neurons in the LHA in the adult mutant. Clearly, both abnormal apoptosis and mis-migration are responsible for the reduction of the Brn2^+^ neurons. Nonetheless, we cannot exclude the possibility that *COUP*-*TFII* gene may also regulate the differentiation of some specific subtypes of PVH neurons, since the expression of *Avp* and *Trh* transcripts is specifically reduced in the mutant (Fig. 6).

In summary, our data provide a functional validation that CNVs of the *COUP*-*TFII* gene are possible causes for growth retardation. In the control mouse embryonic hypothalamus, *COUP*-*TFII* is required to activate or maintain the expression of *Bdnf* and *Nrp1*, which ensure appropriate morphogenesis and function of the HP axis, especially the PVH nucleus, to coordinate the normal growth. In the mutant, the loss of *COUP*-*TFII* results in reduced expression of *Bdnf*, *Nrp1* and *Avp*, which may cause apoptosis and mis-migration of the Brn2^+^ neurons, hypocelluar PVH nucleus, hypoplastic anterior pituitary with blood cell clusters and shrunken posterior pituitary lacking AVP/OT neural innervations. The defective formation of the HP axis, especially the anterior pituitary, leads to growth deficit (Fig. 7C).

Our findings together with other clinical and mouse studies, reveal that *COUP*-*TFII* gene mutations are strongly associated with growth retardation, CHD, CDH, congenital coloboma, and postnatal viability^27,28,34–37,51,60,61^. Furthermore, enhanced expression of *COUP*-*TFII* is correlated with dilated cardiomyopathy^62^ as well as the recurrence and progression of prostate cancer^63^. Thus, *COUP*-*TFII* mutations should be included in the differential diagnosis of birth defects, CHD, CDH, ocular defects and cancer. Our study will benefit the prediction, prevention and treatment of human diseases.

## Methods

### Ethics statement

All animal experiments were carried out following protocols approved by the Animal Ethics Committee of the Shanghai Institute of Biochemistry and Cell Biology. We confirm that all methods were performed in accordance with the relevant guidelines and regulations.

### Animals

*COUP*-*TFII*-*floxed* mice and *RXCre* mice used in the study were of the C57B6/129 mixed background. Male mice were used in body weight study. Noon of the day of vaginal plugs was designated as embryonic day 0.5 (E0.5).

### Hematoxylin and eosin (H&E) staining, Nissl staining, immunofluorescent staining, and immunohistochemical staining

For H&E staining, paraffin sections on slides were dewaxed in xylene for 3 min for three times, and rehydrated in 100% ethanol three times, 95% and 70% ethanol once, with 1 min each. The slides were rinsed in the distilled water, and were stained in hematoxylin solution for 30 sec. Then the slides were washed in running tap water for 2 min. The slides were immersed in acid alcohol for decolorizing, and then rinsed in distilled water. The slides were immersed in Lithium Carbonate solution, and also washed in distilled water. The slides were counterstained in eosin solution for 10 sec. And then, the slides were dehydrated with 95% ethanol once, and 100% ethanol three times with 1 min each. The slides were cleared in xylene twice with 2 min each. The slides were mounted in fume hood. Nissl staining was conducted with 0.1% Cresyl Violet for 10 minutes.

For immunofluorescence staining, the slides were dewaxed and rehydrated the same as H&E staining. Then the slides were washed in distilled water and 1X phosphate buffer solution (PBS) with 1 min each. The slides were treated with boiling 1X antigen retrieval solution (DAKO) for 15 min. After cooling down to room temperature (RT), the slides were rinsed with 1XPBS for 10 min for 3 times. The slides were treated with 3% H_2_O_2_ in 1XPBS for 30 min, and then washed with 1XPBS for 10 min for 3 times. The sections were blocked with buffer containing 1% BSA, 5% Serum in 1XPBS for 1 h at RT, and then incubated with primary antibody in hybridization buffer overnight at 4 °C. After being washed with 1XPBS for 10 min for 3 times, sections were incubated with secondary antibody for 1 h at RT. The sections were washed and counterstained with 4′,6′-diamidino-2-phenylindole (DAPI). After wash, the slides were mounted with mounting medium. In case, TSA kit (Invitrogen) was used. The samples were treated the same as the regular immunofluorescence staining till the completion of the primary antibody incubation. And then the processes were carried out with TSA kit by following the manufactory’s protocol.

For immunohistochemical staining, the sections were treated the same as the immunofluorescence staining till the incubation with the second antibody. The slides were incubated with biotinylated secondary antibody for 1 h at RT. After being washed in 1XPBS for 5 min for 3 times, the slides were incubated with ABC Reagent (Vectorlabs) for 30 min. After being washed in 1XPBS for 5 min for 3 times, the slides were incubated with fresh prepared DAB substrate solution (Vectorlabs). The reaction was terminated till desire signals observed. The slides were counterstained with hematoxylin solution, and then dehydrated, cleared and mounted as described in the H&E staining.

The following primary antibodies were used in the study: rabbit anti-AVP (1:4000, PenisulaLab), rabbit anti-BDNF (1:100, Santa Cruz), rabbit anti-Brn2 (1:400, Santa Cruz), rabbit anti-cleaved-Caspase3 (1:400, Cell Signaling), mouse anti-COUP-TFII (1:500, R&D), goat anti-β-galactosidase (LacZ) (1:400, Biogenesis), rabbit anti-Ki67 (1:400, BD Biosciences), rabbit anti-MCH (1:800, Phoenix Pharm), rabbit anti-Orexin B (1:800, PenisulaLab). The following secondary antibodies were used in the study: donkey anti-mouse IgG biotin-conjugated (1:400, JacksonImmuno); donkey anti-rabbit IgG biotin-conjugated (1:400, Jacksonimmuno); donkey anti-goat IgG biotin-conjugated (1:400, JacksonImmuno); donkey anti-rabbit IgG Alexa-594 (1:400, Invitrogen); donkey anti-goat IgG Alexa-594 (1:400, Invitrogen).

### RNA isolation and quantitative real-time PCR

Total RNAs were prepared from the ventral forebrain of the control and the mutant embryos at E14.5 respectively with TRIzol Reagent (Invitrogen) by following the manufactory’s protocol. Transverse-transcription PCR and quantitative real-time PCR assays were carried out as described previously^39,60^. The universal probe library (Roche) was used in the study. A *student*’*s t*-*test* was used to compare the means of the relative mRNA levels between the control group and the mutant group. Primer sequences and probes are, *Avp*-*f*, *5*′-*ctacgctctccgcttgtttc*-*3*′, *Avp*-*r*, *5*′-*gggcagttctggaagtagca*-*3*′, *Probe #40*; *Arnt2*-*f*, *5*′-*aaacgcataccccagtcttg*-*3*′, *Arnt2*-*r*, *5*′-*cgccactctgtccactctc*-*3*′, *Probe #109*; *Bdnf*-*f*, *5*′-*agtctccaggacagcaaagc*-*3*′, *Bdnf*-*r*, *5*′-*tgcaaccgaagtatgaaataacc*-*3*′, *Probe #31*; *Brn2*-*f*, *5*′-*catcagtggaactagatggacct*-*3*′, *Brn2*-*r*, *5*′-*cttttctgaaggtcccaggtt*-*3*′, *Probe #53*; *Cntf*-*f*, *5*′-*gacctgactgctcttatggaatct*-*3*′, *Cntf*-*r*, *5*′-*gcctggaggttctcttgga*-*3*′, *Probe #13*; *Coup*-*tfI*-*f*, *5*′-*caaagccatcgtgctattca*-*3*′, *Coup*-*tfI*-*r*, *5*′-*cctgcaggctttcgatgt*-*3*′, *Probe #89*; *Coup*-*tfII*-*f*, *5*′-*cctcaaagtgggcatgagac*-*3*′, *Coup*-*tfII*-*r*, *5*′-*tgggtaggctgggtaggag*-*3*′, *Probe #36*; *Crh*-*f*, *5*′-*gaggcatcctgagagaagtcc*-*3*′, *Crh*-*r*, *5*′-*tgttaggggcgctctcttc*-*3*′, *Probe #34*; *Nrp1*-*f*, *5*′-*ccacacacagtgggcttg*-*3*′, *Nrp1*-*r*, *5*′-*ggtccagctgtaggtgcttc*-*3*′, *Probe #26*; *Nrp2*-*f*, *5*′-*ggcttctccgcacgttacta*-*3*′, *Nrp2*-*r*, *5*′-*aaagggacattgcactgaaaa*-*3*′, *Probe #92*; *Ot*-*f*, *5*′-*cacctacagcggatctcagac*-*3*′, *Ot*-*r*, *5*′-*cgaggtcagagccagtaagc*-*3*′, *Probe #27*; *Otp*-*f*, *5*′-*ccagcacagctcaacgaa*-*3*′, *Otp*-*r*,*5*′-*tgaagatgtcggggtagtga*-*3*′, *Probe #55*; *Sim1*-*f*, *5*′-*actcggctctcatctactcca*-*3*′, *Sim1*-*r*, *5*′-*tgaaatgtacatgatcttcccatc*-*3*′, *Probe #49*; *Trh*-*f*, *5*′-*tgcagagtctccaccttgc*-*3*′, *Trh*-*r*, *5*′-*ggggataccagttagcacga*-*3*′, *Probe #21*.

### Chromatin immunoprecipitation (ChIP) and real-time quantitative PCR (qPCR)

Chromatins were prepared from the hypothalamus of mouse embryos at E14.5. *ChIP* assays were carried out with an EZ ChIP Chromatin Immunoprecipitation Kit (Millipore) by following the manufactory’s protocol. The following qPCR assays were performed as described previously^39^. Mouse anti-COUP-TFII antibody, rabbit anti-Sp1 antibody (Millipore), normal mouse IgG and normal rabbit IgG were used in the study. Primer sequences are, a, *5*′-*aagccgcaaagaaggtaagc*-*3*′; b, *5*′-*ctccacttcctcctctccac*-*3*′; c, *5*′-*ctgtaccccaagacctctga*-*3*′; d, *5*′-*ccatccatgaattagccagca*-*3*′.

## Electronic supplementary material

Supplementary file

## References

[1] Morton GJ, Cummings DE, Baskin DG, Barsh GS, Schwartz MW (2006). Central nervous system control of food intake and body weight. Nature.

[2] Morton GJ, Meek TH, Schwartz MW (2014). Neurobiology of food intake in health and disease. Nature reviews. Neuroscience.

[3] Gautron L, Elmquist JK, Williams KW (2015). Neural control of energy balance: translating circuits to therapies. Cell.

[4] Yaswen L, Diehl N, Brennan MB, Hochgeschwender U (1999). Obesity in the mouse model of pro-opiomelanocortin deficiency responds to peripheral melanocortin. Nature medicine.

[5] Ollmann MM (1997). Antagonism of central melanocortin receptors *in vitro* and *in vivo* by agouti-related protein. Science.

[6] Graham M, Shutter JR, Sarmiento U, Sarosi I, Stark KL (1997). Overexpression of Agrt leads to obesity in transgenic mice. Nature genetics.

[7] Huszar D (1997). Targeted disruption of the melanocortin-4 receptor results in obesity in mice. Cell.

[8] Fan W, Boston BA, Kesterson RA, Hruby VJ, Cone RD (1997). Role of melanocortinergic neurons in feeding and the agouti obesity syndrome. Nature.

[9] Balthasar N (2005). Divergence of melanocortin pathways in the control of food intake and energy expenditure. Cell.

[10] Atasoy D, Betley JN, Su HH, Sternson SM (2012). Deconstruction of a neural circuit for hunger. Nature.

[11] Shimada M, Tritos NA, Lowell BB, Flier JS, Maratos-Flier E (1998). Mice lacking melanin-concentrating hormone are hypophagic and lean. Nature.

[12] Sakurai, T. *et al*. Orexins and orexin receptors: a family of hypothalamic neuropeptides and G protein-coupled receptors that regulate feeding behavior. *Cell* **92**, 1 page following 696 (1998). 10.1016/s0092-8674(02)09256-5 9527442

[13] Hara J (2001). Genetic ablation of orexin neurons in mice results in narcolepsy, hypophagia, and obesity. Neuron.

[14] Scott LV, Dinan TG (1998). Vasopressin and the regulation of hypothalamic-pituitary-adrenal axis function: implications for the pathophysiology of depression. Life sciences.

[15] Cunningham ET, Sawchenko PE (1991). Reflex control of magnocellular vasopressin and oxytocin secretion. Trends in neurosciences.

[16] Swanson LW, Sawchenko PE (1983). Hypothalamic integration: organization of the paraventricular and supraoptic nuclei. Annual review of neuroscience.

[17] Nakai S (1995). The POU domain transcription factor Brn-2 is required for the determination of specific neuronal lineages in the hypothalamus of the mouse. Genes & development.

[18] Schonemann MD (1995). Development and survival of the endocrine hypothalamus and posterior pituitary gland requires the neuronal POU domain factor Brn-2. Genes & development.

[19] Michaud JL, Rosenquist T, May NR, Fan CM (1998). Development of neuroendocrine lineages requires the bHLH-PAS transcription factor SIM1. Genes & development.

[20] Acampora D (1999). Progressive impairment of developing neuroendocrine cell lineages in the hypothalamus of mice lacking the Orthopedia gene. Genes & development.

[21] Hosoya T (2001). Defective development of secretory neurones in the hypothalamus of Arnt2-knockout mice. Genes to cells: devoted to molecular & cellular mechanisms.

[22] Michaud JL, DeRossi C, May NR, Holdener BC, Fan CM (2000). ARNT2 acts as the dimerization partner of SIM1 for the development of the hypothalamus. Mechanisms of development.

[23] Wang W, Lufkin T (2000). The murine Otp homeobox gene plays an essential role in the specification of neuronal cell lineages in the developing hypothalamus. Developmental biology.

[24] Keith B, Adelman DM, Simon MC (2001). Targeted mutation of the murine arylhydrocarbon receptor nuclear translocator 2 (Arnt2) gene reveals partial redundancy with Arnt. Proceedings of the National Academy of Sciences of the United States of America.

[25] Nakamura E (2011). 5.78 Mb terminal deletion of chromosome 15q in a girl, evaluation of NR2F2 as candidate gene for congenital heart defects. European journal of medical genetics.

[26] Rudaks LI, Nicholl JK, Bratkovic D, Barnett CP (2011). Short stature due to 15q26 microdeletion involving IGF1R: report of an additional case and review of the literature. American journal of medical genetics. Part A.

[27] Slavotinek AM (2006). Array comparative genomic hybridization in patients with congenital diaphragmatic hernia: mapping of four CDH-critical regions and sequencing of candidate genes at 15q26.1-15q26.2. European journal of human genetics: EJHG.

[28] Klaassens M (2005). Congenital diaphragmatic hernia and chromosome 15q26: determination of a candidate region by use of fluorescent *in situ* hybridization and array-based comparative genomic hybridization. American journal of human genetics.

[29] Biggio JR, Descartes MD, Carroll AJ, Holt RL (2004). Congenital diaphragmatic hernia: is 15q26.1-26.2 a candidate locus?. American journal of medical genetics. Part A.

[30] Arrington CB (2012). A family-based paradigm to identify candidate chromosomal regions for isolated congenital diaphragmatic hernia. American journal of medical genetics. Part A.

[31] Roback EW (1991). An infant with deletion of the distal long arm of chromosome 15 (q26.1—qter) and loss of insulin-like growth factor 1 receptor gene. American journal of medical genetics.

[32] Tsai SY, Tsai MJ (1997). Chick ovalbumin upstream promoter-transcription factors (COUP-TFs): coming of age. Endocrine reviews.

[33] High FA (2016). De novo frameshift mutation in COUP-TFII (NR2F2) in human congenital diaphragmatic hernia. American journal of medical genetics. Part A.

[34] Kantarci S, Donahoe PK (2007). Congenital diaphragmatic hernia (CDH) etiology as revealed by pathway genetics. American journal of medical genetics. Part C, Seminars in medical genetics.

[35] Al Turki S (2014). Rare variants in NR2F2 cause congenital heart defects in humans. American journal of human genetics.

[36] Pereira FA, Qiu Y, Zhou G, Tsai MJ, Tsai SY (1999). The orphan nuclear receptor COUP-TFII is required for angiogenesis and heart development. Genes & development.

[37] You LR (2005). Mouse lacking COUP-TFII as an animal model of Bochdalek-type congenital diaphragmatic hernia. Proceedings of the National Academy of Sciences of the United States of America.

[38] Swindell EC (2006). Rx-Cre, a tool for inactivation of gene expression in the developing retina. Genesis.

[39] Tang K, Rubenstein JL, Tsai SY, Tsai MJ (2012). COUP-TFII controls amygdala patterning by regulating neuropilin expression. Development.

[40] Muller EE, Locatelli V, Cocchi D (1999). Neuroendocrine control of growth hormone secretion. Physiological reviews.

[41] Kappeler L (2008). Brain IGF-1 receptors control mammalian growth and lifespan through a neuroendocrine mechanism. PLoS biology.

[42] Kappeler L (2009). Early postnatal nutrition determines somatotropic function in mice. Endocrinology.

[43] Sabra-Makke L (2013). Hypothalamic ventromedial COUP-TFII protects against hypoglycemia-associated autonomic failure. Proceedings of the National Academy of Sciences of the United States of America.

[44] Enderlin S, Norman AW, Celio MR (1987). Ontogeny of the calcium binding protein calbindin D-28k in the rat nervous system. Anatomy and embryology.

[45] Alvarez-Bolado G, Rosenfeld MG, Swanson LW (1995). Model of forebrain regionalization based on spatiotemporal patterns of POU-III homeobox gene expression, birthdates, and morphological features. The Journal of comparative neurology.

[46] Chao MV (2003). Neurotrophins and their receptors: a convergence point for many signalling pathways. Nature reviews. Neuroscience.

[47] Park H, Poo MM (2013). Neurotrophin regulation of neural circuit development and function. Nature reviews. Neuroscience.

[48] Ip NY, Yancopoulos GD (1996). The neurotrophins and CNTF: two families of collaborative neurotrophic factors. Annual review of neuroscience.

[49] Xu B (2003). Brain-derived neurotrophic factor regulates energy balance downstream of melanocortin-4 receptor. Nature neuroscience.

[50] Pipaon C, Tsai SY, Tsai MJ (1999). COUP-TF upregulates NGFI-A gene expression through an Sp1 binding site. Molecular and cellular biology.

[51] Tang K, Tsai SY, Tsai MJ (2015). COUP-TFs and eye development. Biochimica et biophysica acta.

[52] Galabov P, Schiebler TH (1978). The ultrastructure of the developing neural lobe. Cell and tissue research.

[53] Abuzzahab MJ (2003). IGF-I receptor mutations resulting in intrauterine and postnatal growth retardation. The New England journal of medicine.

[54] Liu JP, Baker J, Perkins AS, Robertson EJ, Efstratiadis A (1993). Mice carrying null mutations of the genes encoding insulin-like growth factor I (Igf-1) and type 1 IGF receptor (Igf1r). Cell.

[55] Holzenberger M (2000). A targeted partial invalidation of the insulin-like growth factor I receptor gene in mice causes a postnatal growth deficit. Endocrinology.

[56] Markakis EA (2002). Development of the neuroendocrine hypothalamus. Frontiers in neuroendocrinology.

[57] Takamoto N (2005). Haploinsufficiency of chicken ovalbumin upstream promoter transcription factor II in female reproduction. Molecular endocrinology.

[58] Lewin GR, Barde YA (1996). Physiology of the neurotrophins. Annual review of neuroscience.

[59] Jones KR, Farinas I, Backus C, Reichardt LF (1994). Targeted disruption of the BDNF gene perturbs brain and sensory neuron development but not motor neuron development. Cell.

[60] Tang K (2010). COUP-TFs regulate eye development by controlling factors essential for optic vesicle morphogenesis. Development.

[61] Yang, X. F., S., Tang, K. COUP-TF genes, human diseases, and the development of the central nervous system in murine models. *Current Topics in Developmental Biology*, doi:10.1016/bs.ctdb.2016.12.002 (2017). 10.1016/bs.ctdb.2016.12.002 28527575

[62] Wu SP (2015). Increased COUP-TFII expression in adult hearts induces mitochondrial dysfunction resulting in heart failure. Nature communications.

[63] Qin J (2013). COUP-TFII inhibits TGF-beta-induced growth barrier to promote prostate tumorigenesis. Nature.

