# Author Correction: Abnormal Paraventricular Nucleus of Hypothalamus and Growth Retardation Associated with Loss of Nuclear Receptor Gene *COUP-TFII*

**DOI:** 10.1038/s41598-018-25537-y

**Published:** 2018-05-11

**Authors:** Su Feng, Can Xing, Tingyu Shen, Yunbo Qiao, Ran Wang, Jun Chen, Jiaoyang Liao, Zhuo Lu, Xiong Yang, Saber Mohamed Abd-Allah, Jinsong Li, Naihe Jing, Ke Tang

**Affiliations:** 1 Institute of Life Science, Nanchang University, Nanchang, Jiangxi 330031 China; 2 State Key Laboratory of Cell Biology, CAS Center for Excellence in Molecular Cell Science, Shanghai Institute of Biochemistry and Cell Biology, Chinese Academy of Sciences, University of Chinese Academy of Sciences, 320 Yue Yang Road, Shanghai, 200031 China; 3 School of Life Science and Technology, ShanghaiTech University, 100 Haike Road, Shanghai, 201210 China; 4 Theriogenology Department, Faculty of Veterinary Medicine, Beni-Suef University, Beni-Suef, 62511 Egypt; 5 Precise Genome Engineering Center, School of Life Sciences, Guangzhou University, Guangzhou, 510006 China

Correction to: *Scientific Reports* 10.1038/s41598-017-05682-6, published online 13 July 2017

In this Article, Affiliation 4 was incorrectly listed as ‘Institute Theriogenology Department, Faculty of Veterinary Medicine, Beni-Suef University, Beni-Suef, 62511, Egypt.’ The correct affiliation is listed below:

Theriogenology Department, Faculty of Veterinary Medicine, Beni-Suef University, Beni-Suef, 62511, Egypt.

