# Supplementary material for: Abnormal Paraventricular Nucleus of Hypothalamus and Growth Retardation Associated with Loss of Nuclear Receptor Gene COUP-TFII
Source: Sci Rep. 2017 Jul 13;7:5282. doi: 10.1038/s41598-017-05682-6 (PMC5509697; doi:10.1038/s41598-017-05682-6)

Abnormal Paraventricular Nucleus of Hypothalamus and Growth Retardation Associated with Loss of Nuclear Receptor Gene *COUP-TFII*

Su Feng[1,2,#], Can Xing[1,#], Tingyu Shen[1,#], Yunbo Qiao[2], Ran Wang[2], Jun Chen[2], Jiaoyang Liao[2], Zhuo Lu[1], Xiong Yang[1], Saber Mohamed Abd-Allah[4], Jinsong Li[2], Naihe Jing[2, 3*], Ke Tang[1,*]

1. Institute of Life Science, Nanchang University, Nanchang, Jiangxi 330031, China,

2. State Key Laboratory of Cell Biology, CAS Center for Excellence in Molecular Cell Science, Shanghai Institute of Biochemistry and Cell Biology, Chinese Academy of Sciences; University of Chinese Academy of Sciences, 320 Yue Yang Road, Shanghai 200031, China,

3. School of Life Science and Technology, ShanghaiTech University, 100 Haike Road, Shanghai 201210, China,

4. Theriogenology Department, Faculty of Veterinary Medicine, Beni-Suef University, Beni-Suef, 62511, Egypt.

# These authors contributed equally to this work.
* To whom the correspondence should be addressed at: Dr. Ke Tang, E-mail: ktang.sc@gmail.com; Dr. Naihe Jing, E-mail: njing@sibcb.ac.cn.

Figure legends

Figure S1. Number of GHRH$^+$ neurons is reduced in the arcuate nucleus of hypothalamus in the *COUP-TFII* mutant. Compared with the control (A,C and inserts), there are much few GHRH$^+$ neurons in the arcuate nucleus of hypothalamus in the *COUP-TFII* mutant at 1M (B,D and inserts), and the reduction is significant (E). Similar as the *RXCre/+;COUP-TFII*$^{F/+}$ control mouse (F, H), the expression of LacZ is barely detected in the arcuate nucleus of hypothalamus in the *RXCre/+;COUP-TFII*$^{F/F}$ mutant mouse at 1M (G,I), indicating *COUP-TFII* gene is not deleted in the arcuate nucleus of hypothalamus in the mutant. 3V, third ventricle; ARC, arcuate nucleus of hypothalamus; ME, median eminence. Scale bars, A-D, F-I, 200 μm; inserts in A-D, 100 μm.

Figure S2. A few Calbindin$^+$ neurons are mis-located laterally to the caudal PVH nucleus in the *COUP-TFII* mutant. The immunofluorescence staining assays with a Calbindin specific antibody were performed with coronal sections at the caudal PVH level of the control and the mutant. Calbindin$^+$ neurons are mainly detected at the PVH nucleus in the control at E15.5 (A,C). Calbindin$^+$ neurons are reduced in the PVH nucleus of the mutant (B,D), instead that compared with the control (E,G), there are more Calbindin$^+$ neurons mis-located laterally to the PVH nucleus of the mutant. PVH, paraventricular nucleus of hypothalamus. Scale bars, A-D, E-H, 100 μm.

Figure S3. There are more cleaved-Caspase-3 labeled apoptotic cells in the

mutant hypothalamus at E13.5. Compared with the control at E13.5 (A,C,E), there are more cleaved Caspase-3 signals in the mutant hypothalamus (B,D,F). C,D, insert in A; D,F, insert in B. PVH, paraventricular nucleus of hypothalamus. Scale bars, A,B, 200 μm; C-F, 200 μm.

Figure S4. COUP-TFII and Sp1 protein are recruited at a conserved Sp1 site of the *Nrp1* gene. A conserved Sp1 site was identified at the intron 12 of the *Nrp1* gene. *ChIP-qPCR* assays with the chromatin prepared from the hypothalamus at E14.5 show that the binding of COUP-TFII and Sp1 is enriched at the conserved Sp1 site of *Nrp1* gene, but not at a negative control non-Sp1 site at 3'UTR region. Ex, exon. The data indicate the mean ± SD. Student t-test, ***, $P<0.005$; †, $P<0.001$. Primers a/b target the Sp1 site, and Primers c/d target the negative control site. Primer sequences are, a, *5'-catagagcgagttccttcca*-3'; b, 5'-*tcttcaagggctttccagtg*-3'; c, 5'-*ctgtggcctcagtacccata*-3'; d, 5'-*tggaggtcacaaacacgaga*-3'.

Figure S5. Expression of both AVP and OT is reduced in the posterior pituitary of the mutant. Compared with the control (A,C), the expression of AVP is barely detected in the posterior pituitary of the mutant at P0 (B,D). Compared with the control (E,G), the expression of OT is hardly detected in the mutant posterior pituitary of the mutant at P0 (F,H). P, posterior pituitary. Scale bar, A-H, 200 μm.

Methods

Immunofluorescence staining and *ChIP-qPCR* assays were conducted the same as those in the main text.

The following primary antibodies were used: rabbit anti-AVP (1:4000, PenisulaLab), rabbit anti-Calbindin (1:400, Spring Bioscience), rabbit anti-cleaved-Caspase3 (1:400, Cell Signaling), mouse anti-GHRH (1:50, Elabscience), goat anti-oxytocin (1:400, PenisulaLab).

Fig. S1. Feng et al.

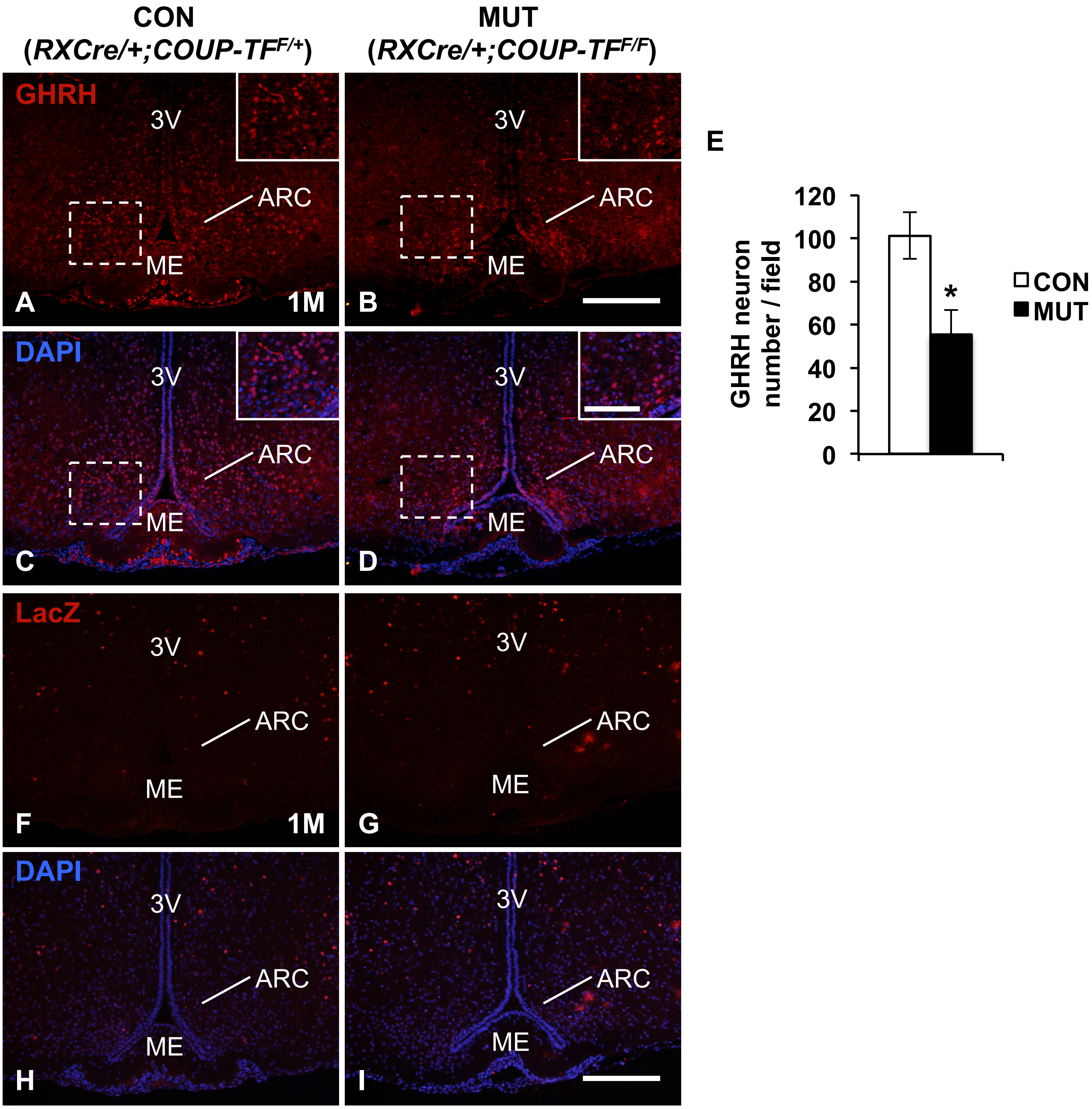

Fig. S2. Feng et al.

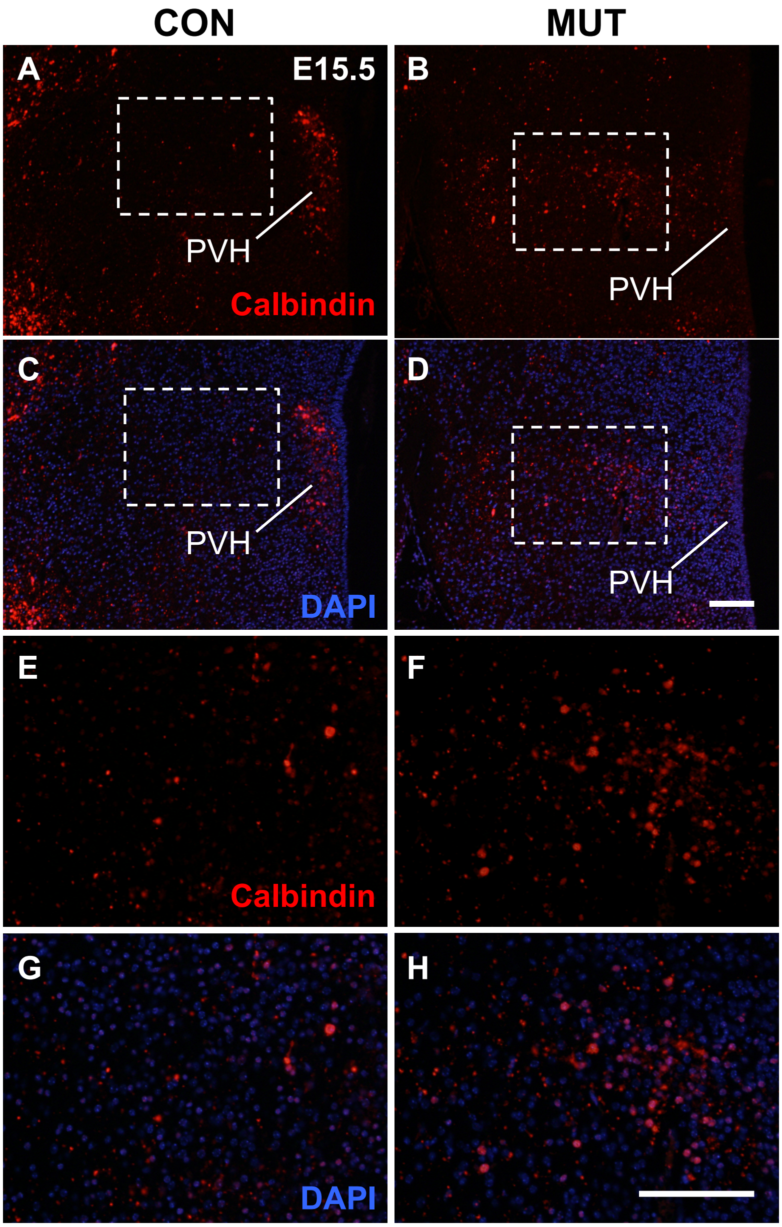

Fig. S3. Feng et al.

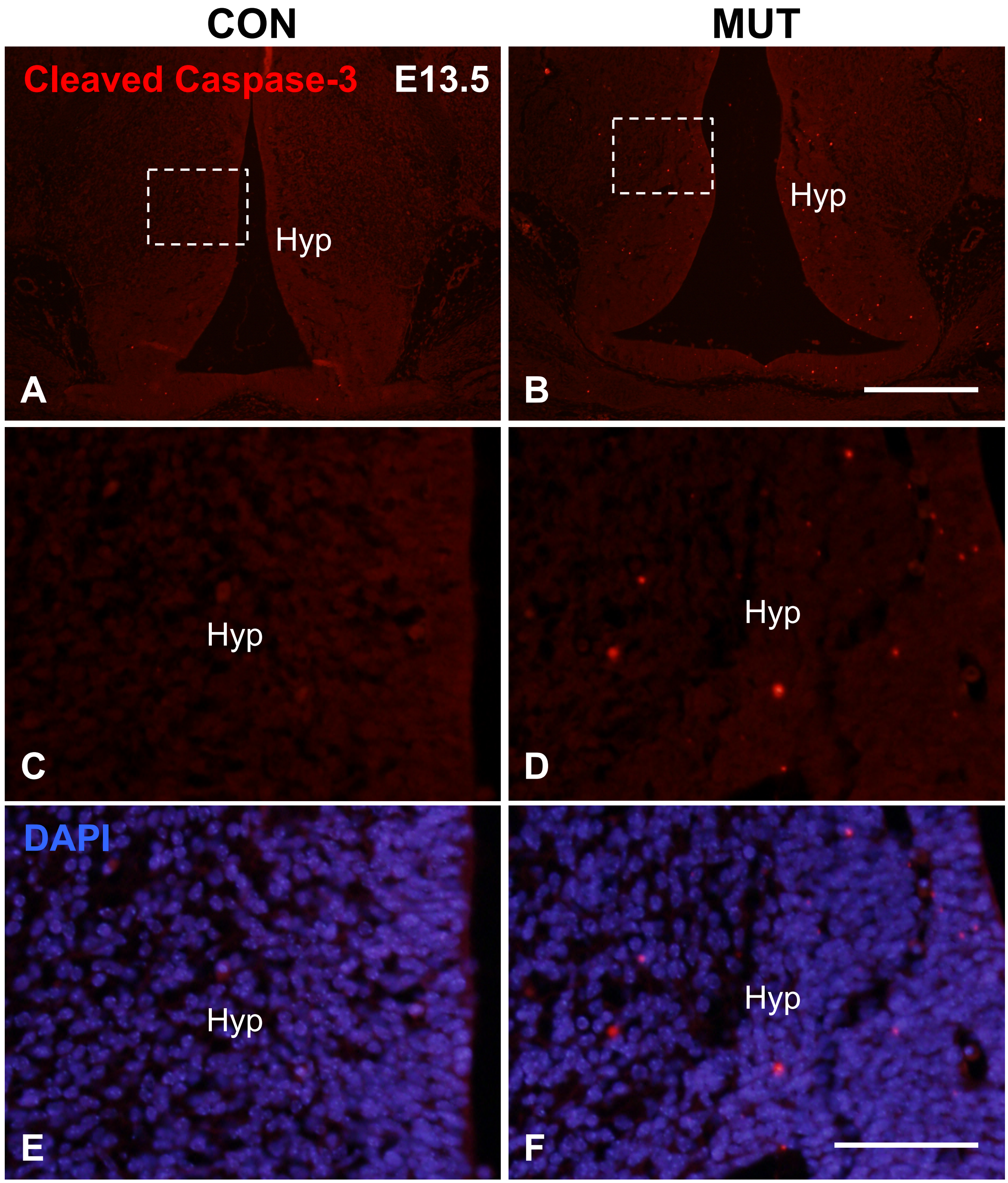

Fig. S4. Feng et al.

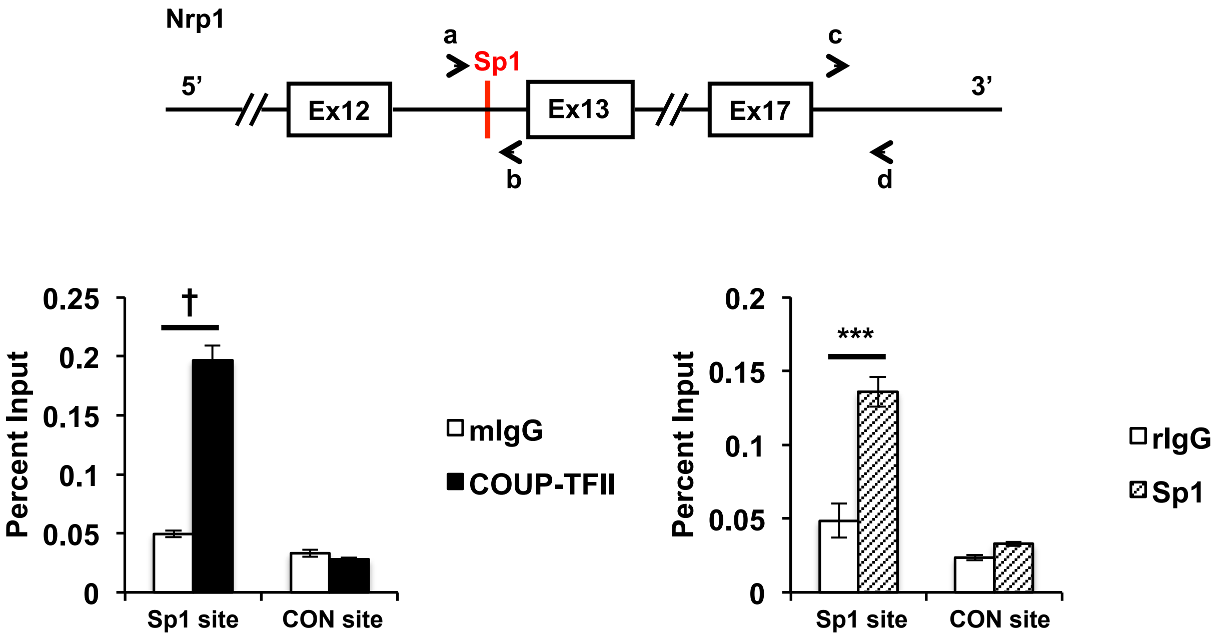

Fig. S5. Feng et al.

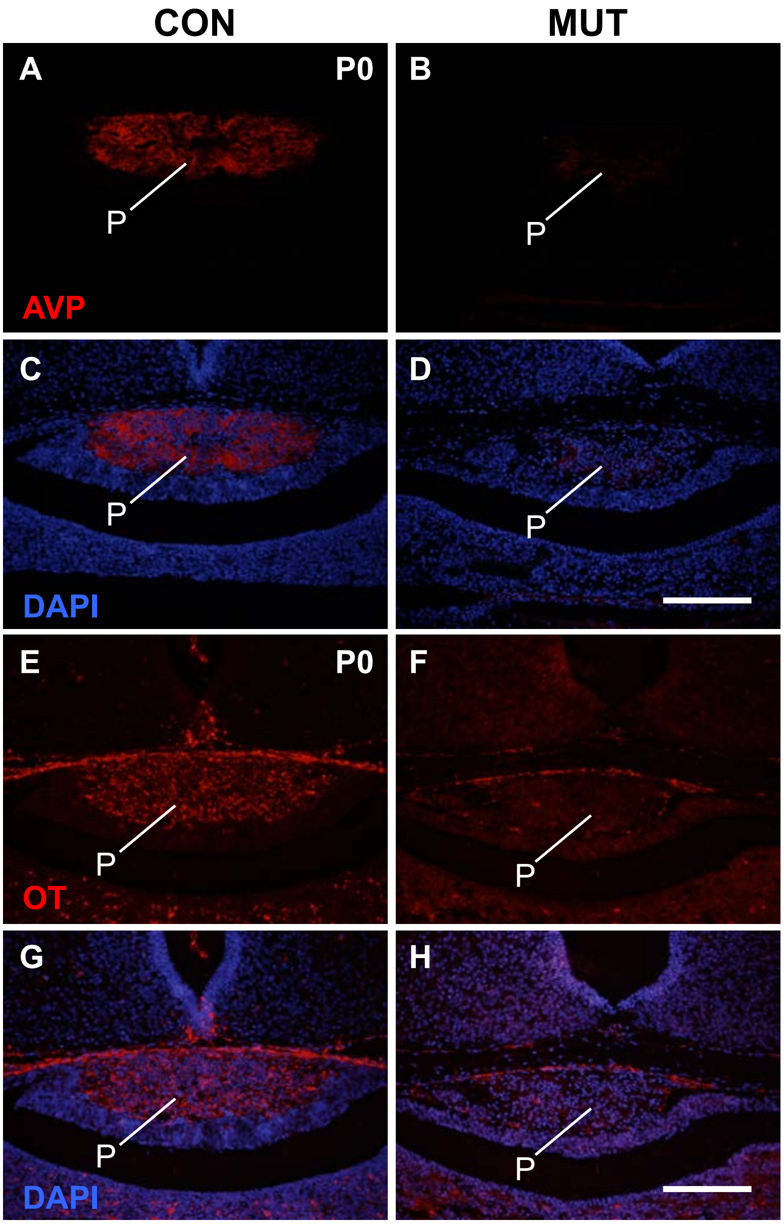

Supplement: Supplementary file 1 — Supplementary file [file 41598_2017_5682_MOESM1_ESM.pdf]
